# Multiple Approaches to the Trophic Role of Mesopelagic Fish around the Iberian Peninsula

**DOI:** 10.3390/ani13050886

**Published:** 2023-02-28

**Authors:** Ainhoa Bernal, Víctor Manuel Tuset, María Pilar Olivar

**Affiliations:** 1 Department of Renewable Marine Resources, Institut de Ciències del Mar (ICM-CSIC), 08003 Barcelona, Spain; 2 Instituto de Oceanografía y Cambio Global (IOCAG), Universidad de Las Palmas de Gran Canaria (ULPGC), Parque Científico Tecnológico Marino de Taliarte, 35214 Las Palmas de Gran Canaria, Spain

**Keywords:** feeding ecology, dietary niche partitioning, stomach contents

## Abstract

**Simple Summary:**

Mesopelagic fish form the largest fish biomass in the oceans. They are essential intermediates in marine food webs, having an important role as zooplankton consumers and prey for top predators such as pelagic fish or marine mammals, as well as in connecting the surface and more impoverished oceanic depths, since many mesopelagic species perform diel vertical migrations. The collapse of most commercial fisheries has led to the consideration of the future commercial exploitation of these fish, which is highly debated. Unfortunately, we have limited information about their feeding strategies and contribution to the marine carbon pump of mesopelagic fish, due to the difficulty of investigating their habitat, the twilight zone. This study analyzed the diets of mesopelagic fish species around the Iberian Peninsula (Atlantic and Mediterranean areas) in terms of numerical prey abundance and carbon weight, which allowed for their assignment to specific feeding guilds. We found that, despite their great diversity, mesopelagic fish food resource use is partitioned by being spatially segregated, using different feeding strategists (generalist, facultative feeding behavior, and specialized diets), or as a result of their dietary shift from juvenile to adult stages, rather than having different food compositions. The assessment of the trophic role of mesopelagic species will provide baseline data regarding the impacts that their potential exploitation might have on the populations of top predators that also play a key role in regulating marine ecosystems.

**Abstract:**

Myctophids, commonly vertical migrators, and partial and non-migrant stomiiforms constitute most of the mesopelagic biomass, and transport organic matter throughout the food web from the surface to the ocean’s depths. Both the diet and trophic structure of twenty-nine species of mesopelagic fish collected around the Iberian Peninsula were studied through the analysis of stomach contents, quantifying food items with a high taxonomic resolution. The investigation covered oligotrophic to productive habitats, with sampling stations distributed in five discrete zones of the western Mediterranean Sea and the northeastern Atlantic Ocean. The geographic environmental conditions, migratory behavior, and species-specific body sizes allowed for the identification of some major feeding patterns for these fish communities. The trophic niche of migrant myctophids showed a high overlap, with copepods as the primary prey category. The diet composition of generalist myctophids (e.g., *Ceratoscopelus maderensis* and *Hygophum benoiti*) reflected the distinct zooplanktonic communities between zones. Large stomiiforms (*Chauliodus* spp., *Sigmops elongatus*) preferred feeding on micronekton, while the smallest stomiiforms (e.g., *Argyropelecus* spp., *Cyclothone* spp., *Vinciguerria* spp.) preyed on copepods and ostracods. Given the relevance of the mesopelagic fish communities for commercially exploited species and, therefore, for maintaining the sustainability of the fisheries’ activity in the zones analyzed, the information provided in the present study is essential for a better understanding of the biology and ecology of these species.

## 1. Introduction

Mesopelagic fishes (MFs) remain for most of their lives in the ocean twilight zone (200–1000 m depth), which contains more fish than almost any other part of the ocean. Thus, they form the largest biomass of teleosteans in the oceans, mainly composed of small and non-commercial species of the orders Myctophiformes and Stomiiformes. They include diverse families (Myctophidae, Gonostomatidae, Phosichthyidae, Sternoptychidae, and Stomiidae), with myctophids possessing the highest species richness (245 species belonging to 33 genera) and gonostomatids having the highest numerical abundance [1,2,3,4,5,6]. Recent studies have estimated the worldwide MF biomass to be around 11–15 Gt [7], but this estimation is still quite imprecise, and scientists are trying to obtain more accurate approximations to robustly report biomass (e.g., objectives of the European project SUMMER (https://summerh2020.eu, accessed on 1 September 2021) and MEESO (https://www.meeso.org, accessed on 1 September 2021). The most recent estimates, combining acoustics and modeling, downscaled this number from 3.8–8.3 billion tons [8] to 2.4 billion tons [9]. Interest in mesopelagic fish species has arisen as they may be a potential resource for a new pelagic fishery [10], contributing to the expansion of fish farming as food [11]. Since 2016, Norway has been developing trial fisheries for mesopelagic fishes, such as *Benthosema glaciale* (Myctophidae) and *Mauloricus muelleri* (Sternopthychidae), along the mid-Atlantic Ridge and in the North Sea [11]. Thus far, there has been no interest in collecting regular and extensive data that allows for a deeper understanding of their ecological role (e.g., energy and organic matter transport vectors, carbon sequestration, and food web position) across the oceans, as has been carried out for commercial species. Thus, there is still great uncertainty regarding the biological and ecological characteristics of many species. Moreover, the difficulty in observing their ecological behavior in situ, at depth (feeding patterns, daily rations, and migratory ascension, etc.), or keeping them alive in the laboratory, and the considerable fishing effort needed to capture them in large numbers, among other factors, are some of the reasons why their biological cycle and ecological role remain poorly understood.

Most myctophids and some stomiiforms undertake diel vertical migrations (DVMs), moving across the water column like hot air balloons from the epipelagic shallow waters where they feed, to deep and less productive oceanic waters, where they rest and excrete organic matter [12,13,14,15,16,17,18,19]. Diel vertical migrations render them energy and organic matter conduits between secondary producers and upper trophic level predators such as pelagic fish, marine mammals, and marine birds, connecting the horizontal strata from surface waters to the depths of the oceans, and in some cases, even reaching the bathyal environment [19,20,21]. The ecological importance of mesopelagic organisms is attributed to the fact that primary production at depths greater than 200 m is negligible, and deep-living fauna depend, for feeding, on the active transport performed by migratory mesopelagic fish, micronekton, and zooplankton [20,21]. These migrants are concentrated in specific water column layers: they remain in the epipelagic stratum at night and are associated with deep scattering layers during the daytime. These distribution patterns have been studied and it has been found that there is a strong correlation with the level of light penetration into the water column, although other environmental factors (i.e., temperature and oxygen) also influence their vertical distributions [22,23]. Moreover, MFs play a major role in the biological carbon pump (i.e., the transformation of CO_2_ and nutrients into organic carbon that sinks to and decomposes in the ocean’s depths), for which they may contribute between 8% and 40% of deep carbon flux, depending on the area [21,24,25,26,27]. The magnitude of the active flux mediated by these fish has been estimated to be comparable to the gravitational flux [27,28,29], but it is still debated if their contribution is large or small [6,21,23]. Their function in reducing CO_2_ emissions (carbon sequestration) is uncertain because we lack the accurate abundance, feeding rate (gut filling and evacuation), and dietary carbon content estimates.

The lack of specific experimental designs focused on obtaining a large collection of MF samples for studying, in depth, their main feeding characteristics and the behavior of each fish species in the mesopelagic community, has resulted in the information being scattered and mostly dedicated to the adult and larval stages versus the transformation and juvenile stages. In addition, the information comes from dispersed locations instead of the study of trophic relationships at the community level. In particular, during transformation (i.e., the transition from larvae to juvenile), fish undergo radical modifications in their morphology and pigmentation, which is associated with changes in their habitat [30,31,32], as well as their feeding habits and trophic niches [33,34,35]. The study of feeding habits and their related physiological changes with development is also a gap in the knowledge of the life cycle of these fish that needs to be filled. Moreover, previous analyses have outlined the existence of differences in feeding strategies and the resource exploitation of spatial niches and foraging areas, particularly between migrant and non-migrant species [36,37]. Mesopelagic fishes vary their feeding strategies and dietary intake as they grow, in association with the development of specific physiological features, morphological variability, or bioluminescent patterns [38]. Some myctophids are mostly defined as generalist zooplanktivores with a diet dominated by mesozooplankton, macrozooplankton, and micronekton [39,40,41,42,43,44]. However, when considering the complete mesopelagic fish community, trophic differences within both prey dietary proportions and exploited trophic niches can be identified among the species [17,43,45]. Extensive literature has been published on the feeding habits and diets of the most abundant species e.g., [45,46,47,48,49,50], but integrative studies analyzing the different components of pelagic food webs and determining the position of MFs at the community level are very limited, e.g., [51]. The combined analysis of stomach contents and biomarkers such as stable isotopes has allowed for the delineation of feeding guilds for several species of the community. Trophic studies based on mesopelagic species are important for unraveling the functioning of open-ocean ecosystems, since myctophids are likely the greatest zooplankton consumers within the midwater fish assemblage, removing a considerable part of its biomass in some regions, e.g., [51,52,53].

The Iberian Peninsula is surrounded by diverse marine ecosystems, from the cold and productive Atlantic waters off the western coast to the oligotrophic and warmer Mediterranean basins. The western Iberian region is affected by annual and seasonal upwelling, and the Gulf of Cadiz is affected by the circulation of the subtropical gyre and currents associated with the continental slope [54,55,56,57,58]. The Mediterranean Sea is characterized by a long period of vertical water stratification, between May and November, and mixing of the water column for the rest of the year, with winter as the most productive season [59]. There is an exchange of water between the Atlantic and the Mediterranean through the Strait of Gibraltar, with an inflow of well-oxygenated water and nutrients from the Atlantic. It is known that the richness of the MF species in the western Mediterranean is lower than that of those in the northeastern Atlantic [5,60,61,62], which is partly due to the poor adaptive capacity of the immigrants to the Mediterranean’s warmer temperature [63,64] and its higher salinity (temperature ca. 13.5 °C and salinity ca. 38.5, below 200 m) [65]. The feeding habits of these species in the western Mediterranean (off the Balearic Islands and the Gulf of Lions) and the northeastern Atlantic (e.g., the northern Mid-Atlantic Ridge, Azores, and the Canary Islands) have been studied through the analysis of stomach contents and stable isotopes at the species and community levels, e.g., [17,42,43,66,67,68,69,70]. These studies found that myctophids have overlapping diet compositions with segregated trophic niches throughout the water column. In addition, they found a variability in feeding times between myctophids, which feed in epipelagic waters at night, and gonostomatids and sternoptychids, which feed intermittently throughout the day near the deep scattering layer. Nevertheless, there are gaps in the knowledge of MF trophic ecology, particularly for species, that inhabit relatively deep waters and exhibit extruded stomachs when sampling nets reach the surface, such as those belonging to the Gonostomatidae, Sternoptychidae, and Stomiidae families. Additional uncertainties include the determination of their daily rations or their feeding guild within the community, among others. The analysis of their diet composition, via two different approaches, and the effect of the environmental factors across species and zones will allow for better insight into the quantitative assessment of feeding habits for trophic model parametrization and fisheries management. Diet compositions and food types in the environment can be modeled in the mesopelagic compartment and can simulate how the food web is driven by environmental variables (temperature and mixing processes, etc.). Fish diets represent an integration of many important ecological components that include behavior, condition, habitat use, energy intake, and inter- and intra-specific interactions, allowing for the understanding of the ecological aspects of these fish species, even at the population and community levels. The current study examines the relative importance of food categories in fish diets, both in terms of the abundance of different prey types and of their overall carbon content, which might be a prerequisite for the estimation of ingestion rates. Accurate data on the carbon content ingested by MF are important, since certain prey types might constitute a resource that is not abundantly consumed but has a high nutritional value. Moreover, carbon mass estimations, combined with the information on DVMs, will be useful for building future models of active carbon transport in oligotrophic and upwelling areas mediated by respiration and excretion.

The present study aims to: (a) characterize the diet composition of 29 species of mesopelagic fish in terms of prey abundance, prey frequency, and prey carbon content, through indexes of relative importance; (b) distinguish the trophic guilds and feeding strategies among mesopelagic fish; (c) establish comparisons between migrant and non-migrant species; and (d) compare the feeding habits of the species in the contrasting areas surrounding the Iberian Peninsula (the western Mediterranean Sea and northeastern Atlantic Ocean), focusing on their particular environmental conditions (thermal and productive regimes) and the diversity of the zooplankton community. We considered the diet composition of the mesopelagic fish community, and migrant and non-migrant species, from the transformation to adult stages.

## 2. Materials and Methods

### 2.1. Study Area

**Figure 1:**
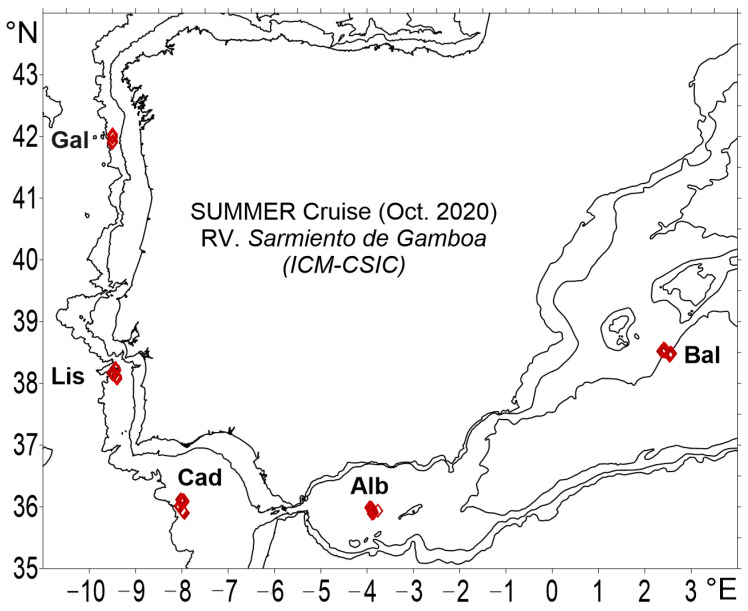
Map of the Iberian Peninsula, surrounded by the western Mediterranean Sea and the northeastern Atlantic Ocean, showing the five sampling areas (Bal: Balearic Sea; Alb: Alboran Sea; Cad: Gulf of Cadiz; Lis: off Lisbon; and Gal: off Galicia). Red diamonds indicate trawling stations with Mesopelagos nets.

Sampling stations were predefined around the Iberian Peninsula within five adjacent zones, all with different environmental conditions and exposed to intense fishing activity. These zones were the Balearic Sea (Bal; general subarea GS5, 38.5° N; 2.4–2.6° W) and Alboran Sea (Alb; GS1, 35.9° N; 3.8–3.9° W) in the western Mediterranean basin, the pelagic waters off the Gulf of Cadiz (Cad; 36° N; 8° W), and Lisbon (Lis; 38.1–38.2° N; 8–9.5° W.) and Galicia (Gal; 42° N; 9.5° W) in the northeastern Atlantic Ocean (Figure 1). The western Mediterranean is a semi-enclosed basin where the number of mesopelagic fish species is much lower than in large oceans [5,62]. The Balearic Sea is characterized by an oligotrophic condition, although the winter mixing of the water column allows for increasing productivity in the following months. However, the survey was run during the oligotrophic season, when the nutrients were depleted in the epipelagic layer after the long summer period of stratification [71,72]. The Alboran Sea is the westernmost Mediterranean sub-basin, connected to the Atlantic Ocean by the Strait of Gibraltar, and is affected by intermittent, enriched deep water upwelling [73]. This enrichment favors the presence of cores with a high phytoplankton and zooplankton abundance [74,75,76,77,78], where the biomass of the zooplankton community is larger in the core of the western anticyclonic gyre (WAG). Our sampling roughly corresponds to the core of the WAG, which tends to have a higher zooplankton biomass than along its edges [73]. In contrast, the Lisbon and Galicia Atlantic stations are much more productive, and the zooplankton composition of these zones is very diverse and abundant, particularly during the summer biomass peak [79]. For the Atlantic side, the survey took place at the end of the western Iberian Peninsula upwelling season [80,81].

### 2.2. Sampling

Fish samples were collected in the Mediterranean Sea and the Atlantic Ocean during the SUMMER survey, run on board the R/V Sarmiento de Gamboa from the 30th of September 2020 to the 18th of October 2020. No commercial species were collected and experiments with living animals were not performed. Fish samples were obtained by using a midwater trawl (Mesopelagos) with a mouth opening of ca. 30 m^2^, and equipped with a graded mesh, from a 30 mm start to a 4 mm end, and SCANMAR and MARPORT sensors [82,83]. The midwater trawl was deployed at 26 stations via oblique hauls at 7 depth layers (depth strata: 0–700 m, 700–600 m, 600–500 m, 500–400 m, 400–300 m, 300–200 m, 200–100 m, and 100–0 m) during the day and night to analyze the diversity and vertical migration patterns. The number of specimens analyzed for the present investigation in each zone and their body size ranges are given in Table 1. Ship speed was kept at 2 knots, and the deployment and retrieval speed of the nets was between 10 and 20 m/min. The mesopelagic fishes were sorted, identified on board, and then frozen (−20 °C). All the fish analyzed for the present investigation were measured in the laboratory to the nearest 0.1 mm of standard length (SL) and weighed (wet weight, WW, and eviscerated WW) to the nearest 0.01 g. The environmental factors used for the subsequent analysis, such as the sea surface temperature and integrated fluorescence (first 200 m of the water column), were obtained with an SBE 911 Plus CTD (Bellevue, WA, USA).

### 2.3. Stomach Content Analyses

A total of 349 specimens from the 29 fish species were dissected to examine their stomach contents. The samples from the different stations and layers from the same zones were combined to ensure a minimum number of individuals per species, zone, and developmental stage (usually, 5 juveniles or adults, and 5 in the transformation stage). The minimum sample size established to run the statistical analysis was not reached for *Photostomias guernei* and *Stomias boa*; however, the diet compositions of both species were described, providing new information on the prey taxa consumed. For the same reason (small sample size), we considered the juvenile and adult fish stages together hereafter. The selection of individuals was performed by prioritizing night trawls between the depths of 0 and 200 m to ensure that most stomachs were full or contained at least one prey item, considering the premise that most mesopelagic fish migrate to the epipelagic layer to forage near the surface at night [5,84,85]. The fishes were thawed, measured for standard length and wet weight, and eviscerated. Afterward, the stomachs were stored in 5% formalin, rinsed, and dissected under stereoscopic microscope for the taxonomic identification of prey items. Each identified prey organism was classified to the lowest taxonomic level allowed by its degree of digestion, then counted and grouped into a functional category. The maximum prey body length and width were measured to the nearest 0.1 µm using a binocular stereoscope (Leica MZ12, Boston, MA, USA) with an ocular micrometric scale. Different reference dimensions, depending on prey taxa, were taken as follows: the standard length for fishes; from the tip of the rostrum to the tip of telson for decapods and euphausiids; from the anterior end of the eyes to the tip of uropods for amphipods; the maximum valve length for ostracods and Bivalvia; the maximum shell length for pteropods; the maximum body length for salps; the trunk length for appendicularians; and the prosome length for copepods. The body dimensions of the identified prey categories were used to estimate the carbon mass, using algorithms compiled from the literature and our own data/calculations (Supplementary Material Table S1). Unlike large commercial fish, mesopelagic species generally prey on tiny organisms that would bias their contribution to the total mass if weighed individually. In these cases, it is desirable to estimate the individual prey weights from direct body length/width measurements using the formulae listed in Table S1. Carbon mass estimations were only carried out when prey items were whole, thus reducing the amount of data available to compare diet compositions in terms of their percentage of carbon mass (%C). For this reason, we did not consider the differences between the zones in this case (except for *Myctophum punctatum*), and described the main dietary outputs for each species by pooling the individual fish from all zones and obtaining the representative carbon weight proportion for each prey taxa and predator species. Rare prey types were either grouped into more general categories, from genus to family classifications, to increase the sample size, or they were included in a group under the designation of “other categories” (categories with <1% abundance in the diet composition) (see Results). In some stomiiforms, stomach content was mainly represented by particulate organic matter (POM) composed of highly heterogeneous components that varied from one sample to another, and, in some cases, was difficult to identify from the very digested remains. These samples were not used for the estimation of carbon due to the difficulty of estimating the C content of the POM and the risk of introducing a large bias (the composition of the POM cannot be determined). To take into account the presence of POM in stomachs, it was considered as one prey item each time it was detected in a fish stomach.

Diet compositions and the importance of each prey category were expressed using different trophic indexes: the percentage frequency of the occurrence of a prey category in the total number of stomachs of a fish species (%F); the percentage number of prey items (%N), the percentage of carbon mass (%C); and two standardized Indexes of Relative Importance (%IRI and %IRIC). The first, %IRI, was calculated as the product of %F per %N, standardized as the percentage of total IRI (%IRI) for each prey item, and the percentage Index of Relative Importance in terms of carbon mass, %IRIC= [(%N + %C)/2] × %F [86], where %C is the relative contribution of each prey in carbon units, was also standardized as the percentage of total IRIC.

### 2.4. Statistical Analyses

Non-metric multidimensional scaling (NMDS) analysis was used to verify the possible dietary differentiation between species and communities based on the mean prey count per species and zone. This ordination technique shows the distance between the considered objects following a previously calculated dissimilarity based on a Bray–Curtis square root transformed matrix. The goodness of fit of the ordination was assessed by the magnitude of the associated stress value, with a value of <0.2, corresponding to a good ordination [87]. A permutational multivariate analysis of variance (PERMANOVA) [88] with a Type III sum of squares was used to test for significant differences in the prey count among species and various influencing factors. The analytical design was composed of fish species (28 levels; random), zones (5 levels: Bal, Alb, Cad, Lis, Gal; random), the developmental stages nested within the species (2 levels: transformation and adult; fixed), and migratory behavior (3 levels; fixed). The migratory behavior of each fish species was defined as migrators (ascending to the first few meters of the water column), partial migrators (up to 100 m in depth), and non-migrators. A similarity percentage analysis (SIMPER) was applied to identify the mean diet dissimilarity between the developmental stages, between the migrant and non-migrant fishes, and between the individuals of the same species but dissimilar zones when significant. Unidentified material, prey categories accounting for less than 1% of the total diet composition, and empty stomachs were deleted from the prey abundance matrix, where the prey numbers of each taxon were considered per fish. The prey taxa were grouped into genus, family, or order for copepods, by the developmental stages in euphausiids, and into broader taxonomic groups for the remaining prey categories. In addition, cluster analyses were performed to assess the degree of similarity in diets (prey count) between the species for each geographical area. A SIMPROF test defined the significant clusters (*p* < 0.05). The significant clusters with >40% of similarity were described in the results section. In all the aforementioned tests, the statistical significance was determined by 999 permutations, and all the analyses were performed in Primer-E v6 + PERMANOVA [89].

To ascertain the intra-specific and ontogenetic (transformation and adult stages) differences in prey number, we considered intervals with a minimum of 5 mm SL and at least 3 prey items per size class. Boxplots were built using the ggstastplot package [90] in R [91]. The advantage of this option was that the comparative analyses based on the analysis of variance (ANOVA) and t-tests were also obtained directly.

The multivariate diet response of each predator species to the explanatory variables of environmental types (the integrated surface fluorescence between the depths of 0 and 200 m (Fl), the sea surface temperature measured at a 5 m depth (SST), and the temperature at a 450 m depth (T450)) was analyzed using a canonical correspondence analysis (CCA) [92] on a matrix of the mean prey abundances per species and zone. The abundance prey matrix was square root transformed to normalize the data and reduce the heteroscedasticity. CCA is a routine used to perform an ordination of the biological assemblages and environmental variables. This routine is constrained to find the linear combinations between the variables that explain the greatest combination of the data cloud. A forward selection was used to rank the environmental variables and test their significance (*p* < 0.05). The coordinate scores associated with each axis provide information on how much the variability inherent in the resemblance matrix is explained by each successive axis. The results of the analysis are shown in an ordination diagram, where the length and direction of each vector indicate the strength and sign of the relationship between that explanatory variable and the axes. The CCA was performed with the software Canoco version 4.55 (Ithaca, NY, USA). All the environmental data were obtained from the CTD measurements throughout the water column.

The diversity of the prey items in the diets was calculated using the Shannon index (H′) and presented as the mean prey diversity per fish species. Only those individuals with at least 50% gut repletion and recognizable prey items were selected for the prey diversity calculations. The prey composition was analyzed here by grouping the species by their vertical migratory behavior, disregarding the zone but not the ontogeny, since even if it is presumed that both the adult and transformation stages of the same species share the same migratory pattern, they might not have the same capability to migrate across the water column and to ingest the same type of prey per size.

## 3. Results

Empty stomachs occurred in 16% of the fish dissected (56 of the 349). A total of 42 prey categories were identified, ignoring the presence of the rare organisms that represented less than 1% of all items in fish stomachs. Copepods were present in the stomachs of most individuals, constituting 19 categories from the 42 identified and occurring in 63% of fish stomachs. Thus, a total 19,136 prey items were identified in the study. Calanoid copepods were the most common prey among myctophids, while they were nearly absent in the stomiid *Chauliodus* spp. The importance of each prey in the species’ diet usually showed great variety, depending on the zone or if it was considered in terms of numerical abundance or carbon weight. The indexes of prey importance are listed in the Supplementary Material Table S2.

### 3.1. Prey Type

#### 3.1.1. Intra-Specific Variability

**Figure 2:**
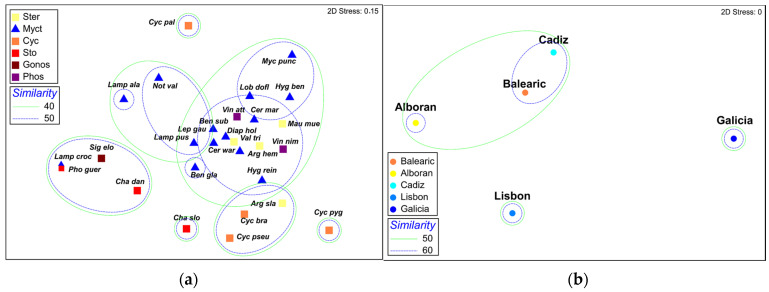
(**a**) Two-dimensional nMDS ordination plot of mesopelagic fish based on prey abundance (%N) and Bray–Curtis distances. Symbols refer to fish orders: triangle: Myctophiformes; and square: Stomiiformes. Colors refer to the 5 fish families: Ster: Sternoptychidae; Myct: Myctophidae; Cyc: only the genus *Cyclothone* within Gonostomatidae; Sto: Stomidae; Gonos: Gonostomatidae; and Phos: Phosycthidae. The species names have been abbreviated using the first three/four letters of the binomial scientific name (e.g., *Myctophum punctatum* has been shortened to *Myc punc*). (**b**) Two-dimensional nMDS plot based on %N and Bray–Curtis distances among zones. The similarity in the diet of species was established at ≥50% and depicted by solid green circles, and ≥60% by dotted blue circles.

The nMDS ordination of the fish species according to the mean prey abundances (Figure 2a) showed a significant segregation of the stomiiforms *Cyclothone braueri*, *C. pygmaea*, *C. pallida*, *C. pseudopallida*, *Chauliodus sloani*, *Chauliodus danae*, and *Sigmops elongatus*. The myctophid *Lampanyctus crocodilus* was grouped with *C. danae*, *Photostomias guernei*, and *S. elongatus,* sharing over 50% of their diet compositions, and the myctophids *L. alatus*, *Notolychnus valdiviae*, and *Lampanyctus pusillus*, shared more than 40% of their diet compositions (*p* = 0.05). The remaining species of myctophids, the two phosyictids, and the three sternoptychids also shared over 40% of their diet compositions. Considering only fish diet similarity among zones (Figure 2b), Galicia is the most dissimilar zone (<30% similarity; *p* = 0.05). However, this dissimilarity is masked by the fact that the number of species collected in this area was low, and they belonged to the stomiiforms *Cyclothone* spp. and *C. danae*, which have already been described as having different feeding compositions. Lisbon was also significantly dissimilar from Cadiz and the Mediterranean zones (49% similarity, *p* = 0.02), the three of which shared >50% similarity (*p* = 0.05).

The PERMANOVA analysis indicated significant differences in the numerical prey abundance among the fish species (Pseudo-F = 4.667, *p* = 0.001), and for species depending on the zone (Pseudo-F = 2.503, *p* = 0.001), their development stage (Pseudo-F = 3.9033, *p* = 0.001), and their migratory behavior (Pseudo-F = 1.887, *p* = 0.02) (Table 2).

At the zonal level, significant differences within species were only detected in some stomiiforms (*Argyropelecus hemigymnus*, *Valenciennellus tripunctulatus*, and *Cyclothone braueri*) and myctophids (*Ceratoscopelus maderensis*, *Diaphus holti*, *Hygophum benoiti*, *Lobianchia dofleini*, and *Myctophum punctatum*) (Table 3). The dominant prey categories (>10% diet contribution) exhibited a greater diet dissimilarity between the zones for each species, while the influence of the remaining categories was less pronounced. These dissimilarities were related to the different genus compositions of each zooplankton community. The main differences between the Alboran Sea and the other study zones were due to the consumption of appendicularians. According to the SIMPER analysis, the diet of *A. hemigymnus* from the Alboran Sea differed from both the Balearic (23%) and Cadiz (18%) zones due to the high contribution of appendicularians in the Alboran zone. The same occurred in the case of *C. maderensis* and *H. benoiti* between the Balearic and Alboran zones, in *M. punctatum* and *L. dofleini* between the Alboran and Lisbon zones, and within *L. dofleini* between the Alboran and Cadiz zones (Table 3). Other important feeding differences among fish species and zones were due to the higher consumption of the calanoid copepod *Centropages* in Lisbon, and also the different contribution of this calanoid to the diet of *C. warmingii* (17%), *L. dofleini* (8%), *M. punctatum* (11%), and *V. tripunctulatus* (10%) in the other zones (Table 3). Other taxa such as *Conchoecia* (ostracod) and *Pleuromamma* (calanoid) were characteristic prey in Cadiz for *C. maderensis*, *D. holti*, *L. dofleini,* and *V. tripunctulatus*. The differences between the zones could be masked by the variability in fish size of each sample cohort per zone, although this only seemed to occur in *L. dofleini*, where the individuals that ate euphausiids corresponded to larger SLs, and those that fed on the non-calanoid copepods (*Oncaea* spp. and Corycaeidae) to smaller SLs.

The diet differences between the transformation and juvenile/adult stages were only obtained for *A. hemigymnus* (*p* = 0.045) and *H. benoiti* (*p* = 0.010) (Table 4). The transformation individuals of *A. hemigymnus* showed a greater within-group diet similarity (ca. 64%) than adults (ca. 31%), capturing mainly ostracods (*Conchoecia* spp.) and non-calanoid copepods (*Oncaea* spp.). In contrast, the adults expanded their diet with appendicularians (19% diet contribution), which might also be influenced by the zone. In *H. benoiti*, the transformation individuals showed a within-group similarity of ca. 41% due to the consumption of oncaeids and calanoids. The adults also presented a similar variability (38%), feeding on mainly copepods of the genera *Pleuromamma* and *Oncaea.*

When the analysis focused on the species’ migration behavior, significant differences were only detected between the migrant and non-migrant species (*t*= 1.32, *p* = 0.007). The dissimilarity between both groups was ca. 85%, with copepods and appendicularians as the most abundant prey in the diets of the migrant fish, although Eucarida (euphausiids and decapods) also influenced this dissimilarity (Table 5).

#### 3.1.2. Community Analyses

**Figure 3:**
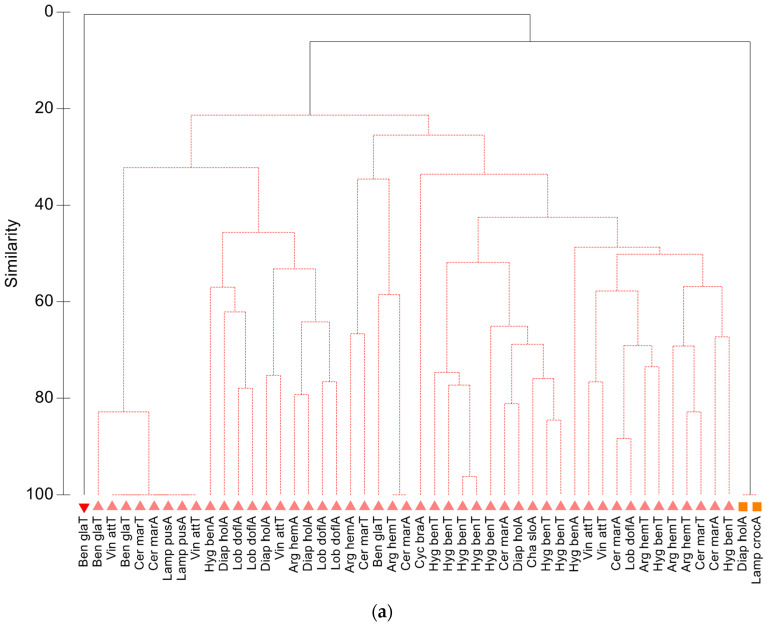

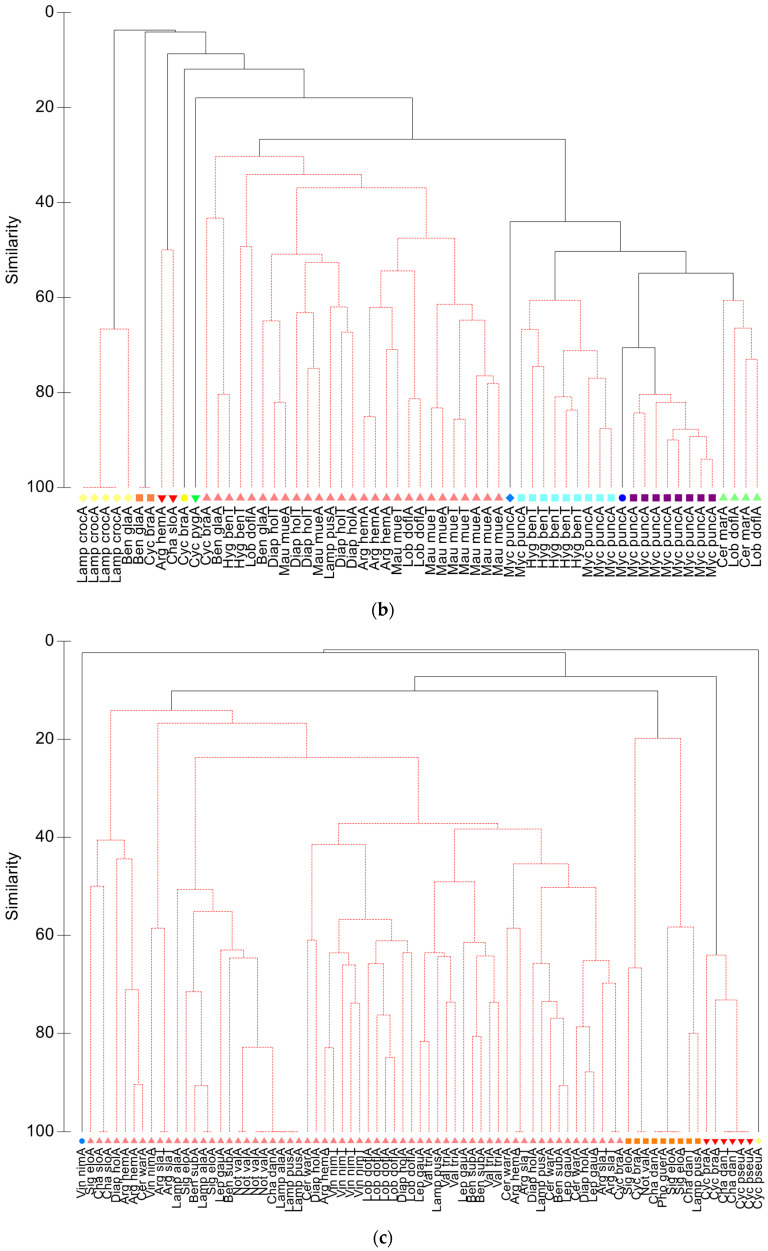

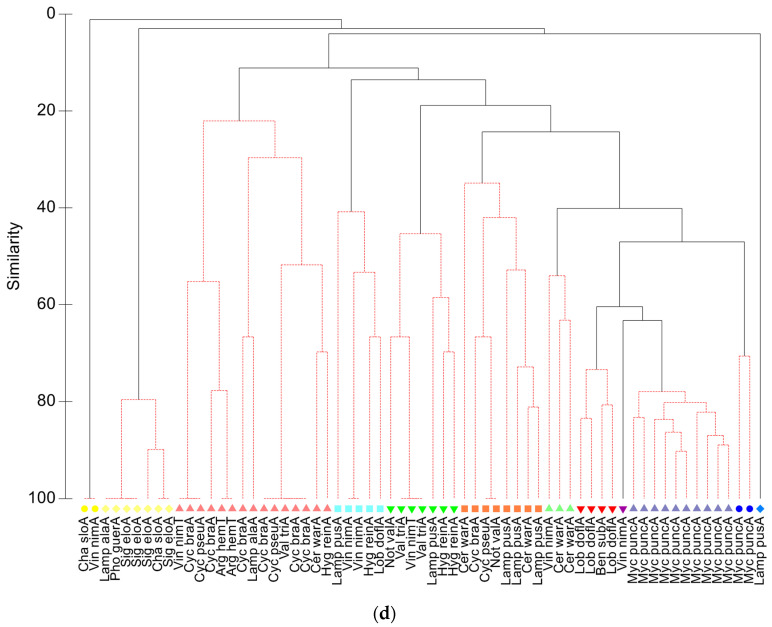
Four dendrograms showing significant groupings for fish diets in each zone, apart from Galicia, after applying cluster analyses to the Bray–Curtis similarity and transformed matrixes of prey abundances. Significant clusters are denoted by colored symbols and black lines (SIMPROF, *p* = 0.05). Dashed lines depict non-significant clusters. Labels indicate the shortened name of the species (first three/four letters of the genus and species) and the developmental attribution: T: transformation; and A: adult (including juveniles). Zones: (**a**) Balearic; (**b**) Alboran; (**c**) Cadiz; and (**d**) Lisbon.

Individual cluster analyses for each zone were performed to establish the fish diet similarities among species. In the Balearic zone, there was only a significant cluster with low similarity (20%), grouping most of the fish together, and only the myctophids *B. glaciale*, *C. maderensis*, and *L. pusillus*, and the phosichthyid *Vinciguerria attenuata*, were non-significantly grouped with >80% similarity (Figure 3a). This result indicates a high individual variability in the diet composition at the species level, revealing the complexity of the community’s food web. A larger number of significant groups were detected in the Alboran Sea. The juvenile and adult individuals of *Lampanyctus crocodilus* shared a 100% diet similarity; *M. punctatum* ca. 70%, and *C. maderensis* and *L. dofleini* a 60% similarity (Figure 3b). Moreover, there was a significant cluster containing the transformation individuals of *H. benoiti* and the adults of *M. punctatum* (60% similarity). Finally, *M. punctatum*, *H. benoiti*, *C. maderensis*, and *L. dofleini* shared a 47% diet similarity.

In the Atlantic waters of the Gulf of Cadiz, three significant groups were detected. The transformation individuals of *Chauliodus danae* and the adults of *Cyclothone braueri* and *C. pseudopallida* shared the highest diet similarity at ca. 60%, while the other clusters contained a mixture of species from different families (Figure 3c). The classification of the Lisbon samples yielded the highest number of significant groups. The juveniles and adults of the stomiids *P. guernei* and *C. sloani*, and the gonostomatid *S. elongatus*, constituted the cluster with the highest diet similarity (80%). There were also other significant clusters composed of the adults of *M. punctatum* (78%), *L. dofleini* (ca. 75%), and *C. maderensis* and *Vinciguerria nimbaria* (ca. 55%). In addition, two other significant clusters (>40% diet similarity) were formed by the mixed species of myctophids and stomiiforms (Figure 3d). The results for the Galicia zone are not presented here, since the number of samples and species for that zone were underrepresented due to the unfavorable conditions for trawling. There were only fish individuals belonging to the genera *Cyclothone* and *Chauliodus*, which is a small representation of the diversity of the mesopelagic fish community. Moreover, the prey contributions to each significant cluster are not described here, as we already described them with the previous SIMPER analysis.

#### 3.1.3. Environmental Influence on Diets

**Figure 4:**
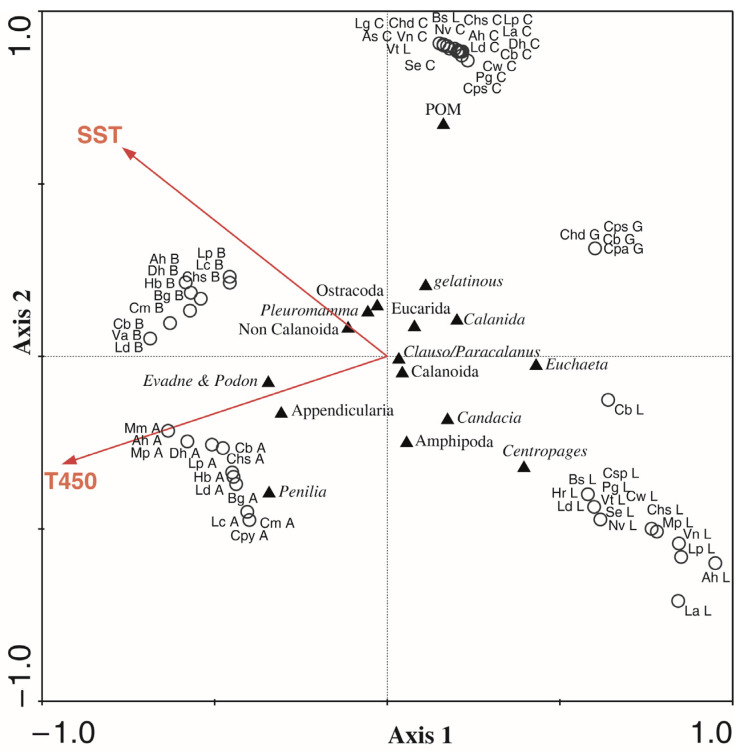
Canonical ordination plot (CCA) for the diet of mesopelagic species. The red arrows indicate the explanatory variables (SST: sea surface temperature; T450: temperature at the 450 m-depth strata; and FI: integrated fluorescence in the first 200 m of the water column). Labels with two or three letters indicate the species name, and the separated upper-case letter indicates the zone (B: Balearic; A: Alboran; C: Cadiz; G: Galicia; and L: Lisbon). Ah: *A. hemigymnus*; BG: *B. glaciale*; Bs: *B. suborbitale*; Cb: *C. braueri*; Cp: *C. pallida*; Cps: *C. pseudopallida*; Cm: *C. maderensis*; Cw: *C. warmingii*; Chs: *C. sloani*; Dh: *D. holti*; Hb: *H. benoiti*; Hr: *H. reinhardtii*; Lc: *L. crocodilus*; Lp: *L. pusillus*; Ld: *L. dofleini*; Mm: *M. muelleri*; Mp: *M. punctatum*; Nv: *N. valdiviae*; Va: *V. attenuata*; Vn: *V. nimbaria*; and Vt: *V. tripunctulatus*.

The CCA ordination for mesopelagic fish diets explained 26% of the total variance of the fish diet–environment dataset. The relationship of the variables T450 and SST with fish diets was significant, while Fl was not (Table 6). The highest contributor to this relationship was T450 (12.6%) and then SST (11.3%). The first canonical axis significantly explained 50% of the species-environment correlation, and the second axis explained 35%. The variable T450 had a negative correlation with both axis 1 (−0.68) and axis 2 (−0.18), showing that the higher temperatures at a 450 m depth (ca. 13.4 °C) explained the diet of the Alboran fishes and the abundance and presence of cladocerans and appendicularians in their stomachs (Figure 4). A lower T450 level (11.3 °C) correlated with the stomiiforms from Galicia that contained a small number of individuals with a differential contribution of undetermined gelatinous prey in *C. pallida*. The sea surface temperature (SST) was also negatively correlated with axis 1 (−0.55), but positively correlated with axis 2 (0.35), explaining that the higher mean surface temperatures (23–24 °C) were related to the diet of the myctophids and stomiiforms from the Balearic Sea (with *Pleuromamma*, non-Calanoida, and Ostracoda as important prey), while the lowest SSTs (ca. 17–18 °C), characteristic of the highly productive Lisbon zone, explained the diet of both myctophids and stomiiforms, with *Centropages* and *Candacia* spp. as the main prey.

### 3.2. Prey Number

**Figure 5:**
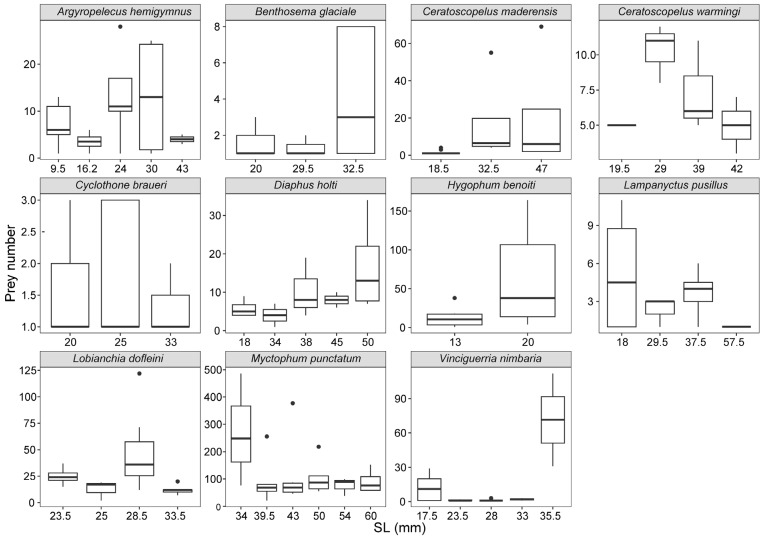
Boxplots for each mesopelagic species showing mean prey number versus standard length (SL (mm)). The line in the box indicates the median value of the data and the upper and lower edges of the box, and the first and third quartiles, respectively. The whiskers extend to 1.5 times the interquartile range. The points outside the ends of the whiskers are outliers. The ticks on the X-axis indicate the end of the size interval for each species. Boxplots for the species are displayed in alphabetic order.

The mean number of prey items (PN) for myctophids (29.9 ± 12.4) was significantly higher (Kruskal–Wallis chi-squared = 16.06, df = 1, *p* = 6.137 × 10^−5^) than for stomiiforms (22.3 ± 7.32). The PN per fish was greatest in *M. punctatum* (486 prey items in an adult with an SL of 34 mm), and the maximum number of taxonomic categories, identified to the lowest taxonomic level, was observed in *L. dofleini* (20 different taxa in an adult with an SL of 27 mm). The highest mean PN per stomach was found in the adults of the myctophid species *M. punctatum* (123 ± 117), *L. dofleini* (29 ± 28), and *C. maderensis* (19 ± 29), and both the transformation and adult individuals of *H. benoiti* (47 ± 56 and 19 ± 2, respectively) (Table S3). Due to the low number of individuals analyzed by size class, no significant intra-specific differences were detected, except for *H. benoiti* (*t*-test = −2.68, *p* = 0.02), which included the transformation stages (<18 mm). Nonetheless, a great variability and low PN values were observed by size class in stomiiforms such as *A. hemigymnus* and *C. braueri* (Figure 5), whereas *V. nimbaria* showed a noticeable increase in PN in larger specimens. A negative trend in the mean PN with size class seemed to occur in *C. warmingii*, *L. dofleini, L. pusillus*, and *M. punctatum*. In contrast, an increase was suggested for *C. maderensis* and *D. holti*.

The species with the highest mean prey diversity (H’ = 1.5−2.0) was *L. dofleini* for all four zones (Table S3). Some fish species such as *B. suborbitale*, *C. warmingii*, *M. punctatum*, and *V. nimbaria* attained H’ values ranging from 1.5 to 1.8, likely reflecting the zonal diversity of the available prey.

### 3.3. Diet Composition

#### 3.3.1. Geographic Analysis

For most MFs, their diet compositions were more varied in the prey taxa in the Atlantic than in the Mediterranean. In the Balearic zone, calanoids represented 59%N (46%N were *Pleuromamma*), 47%, and 46%N of the diet of the myctophids *D. holti*, *H. benoiti,* and *L. dofleini*, respectively. Among the stomiiforms, the *V. attenuata* also fed predominantly on calanoids (62%N, of which 26% were *Pleuromamma*). Small copepods (cyclopoids), mainly comprising the genera *Oncaea*, *Corycaeus*, and *Farranula*, were the most abundant prey items for the myctophids *H. benoiti* (34%), *C. maderensis* (26%), and *D. holti* (15%), as well as for stomiiforms such as *C. sloani* (61.5%), *V. nimbaria* (46%), and *A. hemigymnus* (24%). Ostracoda was the second most abundant prey item in the Balearic Sea for the species *A. hemigymnus* (31%), *V. attenuata* (26%), and *V. nimbaria* (25%). Euphausiids represented 18% in both *D. holti* and *L. dofleini*. (Table S2).

In the Alboran zone, appendicularians were the most abundant prey type for the myctophid species *M. punctatum* (95%), *H. benoiti* (70%), *C. maderensis* (57%), and *L. dofleini* (23%), and for the sternoptychid *A. hemigymnus* (32%). Calanoids were important for *D. holti* (58%, of which 48.5% were *Pleuromamma*), *B. glaciale* (47%), *L. dofleini* (39.2%), and *L. pusillus* (Balearic and Alboran zones together equaled 27%, of which 20% were *Pleuromamma*), and for the small-sized sternoptychids *A. hemigymnus* (52%) and *M. muelleri* (28%). Other abundant prey included cyclopoids for *D. holti*, *B. glaciale*, and *M. muelleri* (21%, 18%, and 39%, respectively). Euphausiids were important for *S. elongatus* (37.5%) and constituted nearly 50%N of the diet of *Lampanyctus crocodilus* from the Balear and Alboran zones (both zones were considered together in this case, because the species sample size per zone was low).

The Gulf of Cadiz seemed to be the zone with the highest prey diversity, as reflected in the fish diets consisting of a mix of various prey in similar proportions, instead of the dominance of particular prey items, except for the genus *Lampanyctus.* Specifically, *Lampanyctus alatus* and *L. pusillus* had diets based mainly on the calanoid copepod *Pleuromamma* (63% and 47% N, respectively) and euphausiids (13% in both species). Both prey categories are vertical migrants that perform displacements all throughout the water column, increasing the likelihood of prey–predator encounters. Copepods were represented in the diets of all fish species, with the most preyed upon categories being the migratory, larger-sized calanoids *Pleuromamma* and *Euchaeta* in *N. valdiviae* (75% and 12.5%, respectively), the calanoids in *B. suborbitale*, *D. holti*, *Cyclothone pseudopallida*, and *C. braueri* (62%, 22%, 60%, and 37.5%, respectively), and the non-calanoids in *L. dofleini* (cyclopoids comprised more than half of its diet, at 54%), and *Argyropelecus sladeni* (21%). Unlike the Mediterranean, ostracods were particularly important in *A. hemigymnus*, *C. warmingii* (19% in both species), *D. holti*, *Hygophum reinhardtii*, and *L. dofleini* (ca. 15% for each of them), and appeared in the stomachs all the fish species in the Gulf of Cadiz aside from *L. alatus*. In *A. hemigymnus*, apart from Ciliophora (a taxon not usually reported as part of its diet, but reaching up to 38%N in one adult individual) and ostracods, cyclopoids were the next most abundant food item (10%). *Ceratoscopelus warmingii* and *D. holti* had diverse diets, with tunicates (appendicularians and salps) constituting 40% and 31% N, respectively (Table S2).

In the Lisbon zone, calanoids and ostracods were, in general, the most abundant prey items in the diets. Ostracods represented relative abundances of 60% for *A. hemigymnus* and 14% for both *C. braueri* and *H. reinhardtii*, while calanoids represented 83%, 75%, 76%, 65%, 38%, and 20% for *L. pusillus*, *N. valdiviae*, *L. dofleini*, *C. warmingii*, *H. reinhardtii*, and *L. alatus*, respectively. The genus *Centropages* represented more than a third of the diet of *L. pusillus* and *C. warmingii*, and 25%, 23%, and 16% of the *N. valdiviae*, *H. reinhardtii*, and *L. dofleini* diets, respectively. Cyclopoids were more abundant in small and medium-sized stomiiforms (*C. braueri*: 57%; and *A. hemigymnus*: 20%) and were also found in small-sized myctophids such as *H. reinhardtii*, *C. warmingii*, *L. dofleini*, and *L. pusillus* (Table S2). The euphausiid abundance in *L. alatus* and *L. pusillus* diets (20% and 7%, respectively) was similar to that observed in the Gulf of Cadiz. *Cyclothone* spp. and *C. danae* were the only species analyzed in Galicia. *Cyclothone braueri* fed on calanoids (50%) and *C. pallida* on appendicularians (70%), while the stomachs of *C. danae* contained decapods (40%) and POM (40%).

Considering a prey’s relative importance to be >10%IRI, copepods had a high importance in the fish diets of all five studied zones, with calanoids more abundant in larger specimens and cyclopoids in smaller-sized ones. Appendicularians were particularly important for some species (*C. maderensis*, *C. warmingii*, *D. holti*, *H. benoiti*, *L. dofleini*, *M. punctatum*, and *A. hemigymnus*) in the Alboran Sea. Euphausiids, however, were linked to species-specific preferences in *L. crocodilus* (89% IRI) and *S. elongatus* (91%), which are large-bodied fishes. Ostracods were the most important prey category consumed by *A. hemigymnus* in the Balearic and Atlantic sites (Balearic: 57.5%; Cadiz: 67%; and Lisbon: 48.5%) and by *Argyropelecus sladeni* in Cadiz (34%), indicating that ostracods are the prey of choice of this genus. In contrast, the presence of ostracods was important in *C. warmingii* (33%), *D. holti* (27.5%), and *L. dofleini* (17%) in Cadiz. It is plausible to think that the differences between both Atlantic zones (Cadiz and Lisbon) could be addressed by the differences in the fish size range.

#### 3.3.2. Species Diet Composition in Terms of Carbon Content

**Figure 6:**
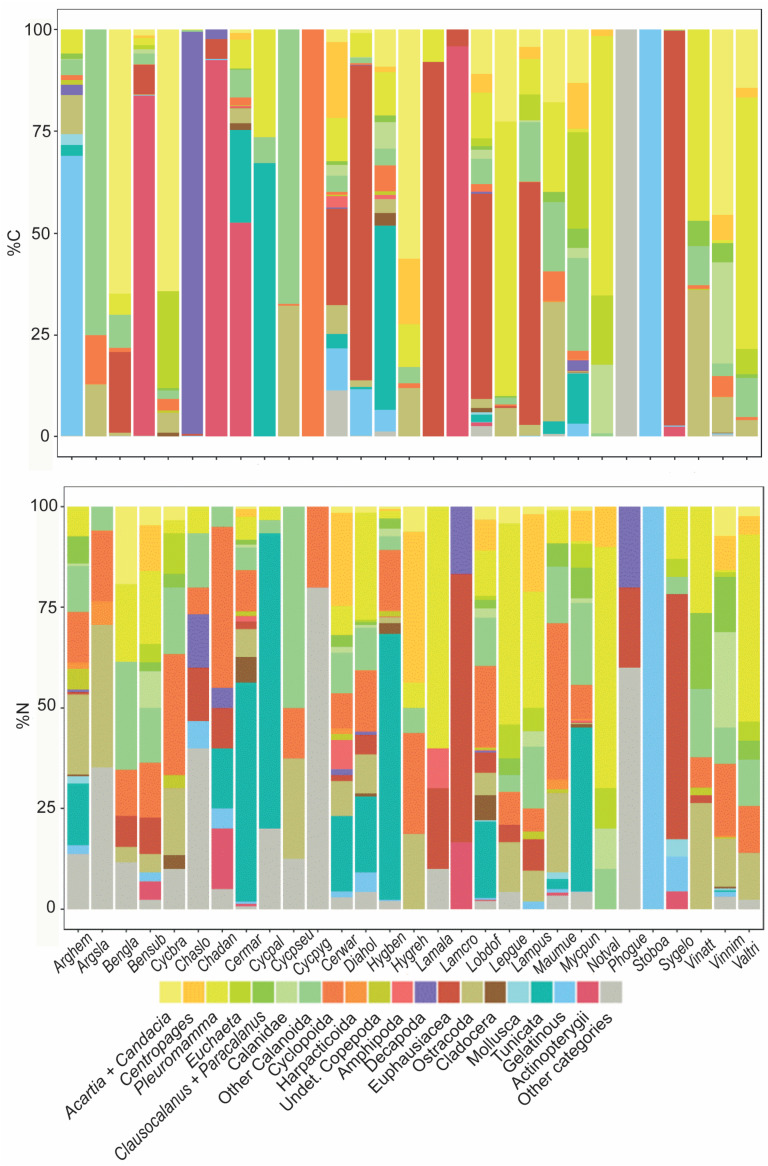
Diet compositions of Myctophiformes and Stomiiformes, pooling individuals of the five zones studied by fish species and expressed as a percentage of carbon content (%C) and percentage of abundance (%N). Prey taxa were reduced to 20 categories; “Other categories” represents <1% N. The names of the fish species are abbreviated as the first three letters of each word of the binomial scientific name.

Among myctophids, calanoids and euphausiids dominated the diet contributions in terms of their carbon content, with different proportions depending on the species. The differences between the congeneric species from the different zones were very noticeable. For example, the diet of *B. glaciale* from the Mediterranean Sea was dominated by calanoids (78% C and IRIC), while the genus *Candacia* constituted 65% of the estimated carbon mass, followed by euphausiids (20% C and 10% IRIC) (Figure 6 and Table S2). In contrast, the *B. suborbitale* inhabiting Atlantic waters preyed on small mesopelagic fish, which were by far their most important dietary item in terms of carbon (84% C and 36% IRIC). The diet of *C. maderensis* (Mediterranean Sea) was composed mainly of fish (53% C and 7.7% IRIC) and tunicates (appendicularians) (23% C and 44.6% IRIC), whereas calanoids (mainly *Centropages*, 19% C, and *Pleuromamma*, 11% C) constituted 40% C (53% IRIC) of the diet of *C. warmingii* (Atlantic waters), followed by euphausiids at 23.6% C (4.3% IRIC), ostracods (13.5% IRIC), and tunicates (15.4% IRIC). The diet of *H. reinhardtii* (Atlantic waters) differed from that of *H. benoiti* (Mediterranean Sea), mainly in its diversity and proportions of calanoids (87% vs. 33% C and 72% vs. 13% IRIC), particularly of the genera *Candacia* and *Centropages* consumed by *H. reinhardtii*. Finally, *L. alatus* and *L. pusillus* showed a similar diet, independent of the zone, mainly based on euphausiids (92% C and 43.5% IRIC; and 60% C and 29% IRIC, respectively), whereas fish constituted most of the dietary carbon in *L. crocodilus* (96% C and 27.3% IRIC).

In *D. holti*, euphausiids (77% C and 27% IRIC) and gelatinous prey (12% C) were important in their diet. In *Lepidophanes gaussi*, *Pleuromamma* represented 68% C (81% IRIC) and *Candacia* represented 23% C (4% IRIC). Euphausiids and calanoids (mainly *Candacia* and *Pleuromamma*) constituted 51% C (30.3% IRIC) and 38% C (40.4% IRIC) of the diet of *L. dofleini*. No dissimilar diet composition was noted at a local scale in *M. punctatum*, which was dominated by tunicates (12.5% C and 26.2% IRIC) and calanoids (79% C and 63.4% IRIC) (Figure 6 and Table S2).

Stomiiforms showed contrasting prey preferences. Large-bodied species such as *C. danae*, *S. elongatus*, and *C. sloani* preyed on large crustaceans, including decapods (99% C and 42% IRIC), euphausiids (97% C and 96% IRIC), and fish (92.5% C and 72.5% IRIC), respectively. Small-bodied species such as *A. hemigymnus*, *A. sladeni*, *M. muelleri*, and *V. tripunctulatus* commonly ingested calanoids (11–95% C) and ostracods (4–36% C), although gelatinous prey (72% C and 27% IRIC) were also important for *A. hemigymnus*. A similar pattern was also recorded for *V. attenuata* (63% C and 77% IRIC for calanoids; and 36% C and 19.5% IRIC for ostracods), whereas *V. nimbaria* fed mainly on calanoids (85% C and 60% IRIC). Conversely, the genus *Cyclothone* showed dissimilar diets among congeneric species, in terms of numerical prey abundance (Figure 6). The diet of *C. braueri* was dominated by copepods, calanoids, and non-calanoids, and that of *C. pallida* and *C. pseudopallida* by tunicates and calanoids, respectively. Notably, the stomachs of *Cyclothone pygmaea* contained mostly dietary remains in an advanced stage of their digestion.

Finally, we think it is worth mentioning the presence of a few pieces of plastic in 3 individuals of *L. pusillus* and *P. guernei*. We found a pink plastic fiber in *L. pusillus*, a non-colored plastic fragment in *P. guernei*, and two plastic fragments (green and black) in the same stomach of another *P. guernei*.

## 4. Discussion

### 4.1. Study Constraints

Dietary studies based on stomach contents can have limitations when trying to ascertain trends in the specific feeding strategies of mesopelagic fish, such as myctophids and stomiiforms, or other pelagic fish. The first limitation is the low number of samples per species due to the time-consuming process of prey identification with this classical method. To provide very specific information at the community level and among these communities is a chimera for many reasons, including monetary funding, the time needed for taxonomic identification, and the increasing need to publish research results quickly. This problem inevitably affects the power of statistical analyses and their conclusions. Even more so, differences in diet compositions are caused by many factors that influence resource partitioning, including the range of vertical distribution, migratory behavior, and feeding strategies [93]. Acknowledging these limitations, in the present study, we provide a general idea of the dietary differences among species, which allows us to establish feeding guilds and relate their dietary preferences to the ecological environment.

The second limitation, which is directly related to the first one, is the view that intra-specific variability is not relevant. Thus, individuals from the same haul should theoretically have similar prey compositions in their diets, since they are aggregated in the same water stratum. One of the most highlighted issues in the present study is precisely the intra-specific variability between and within the size ranges for many species, far from the regional dissimilarity described for some species. This is not novel [43,46], and the problem lies again in the difficulty of providing an accurate picture at the level of community. In this context, our study did not allow for intra- and inter-specific comparisons of some species of stomiids, such as *Photostomias guernei* and *Stomias boa*, due to the low number of specimens collected (only 4 and 2, respectively). The main reason is that both species inhabit very deep waters, ranging from below a depth of 1000 m to below the epipelagic layer (up to a 100 m depth), and are found in low abundances [94,95]. There is a great difficulty in sampling deep water species in sufficiently large numbers to have an adequate sample size for statistical analysis. Despite this, we considered that it is advisable to include them to determine their role in the whole mesopelagic fish community.

Other issues are related to a lack of consensus regarding the method of quantification and determination of the importance of diet composition. Here, we included a method of reconstructing prey carbon mass, usually using body length, similar to that employed by various authors e.g., [26,35,96,97,98]. This is an interesting approximation of diet composition in terms of its application to biomass or energy flow, even when some biases need to be assumed with an underestimation of rapidly digested prey items, in contrast to slowly digested ones. Additionally, it is impossible to quantify the contribution of the POM by this method, which is quite important in deep-living species.

### 4.2. Feeding Guilds

Our findings asserted that the principal food for the most common and frequent fish in the mesopelagic assemblage, in both the western Mediterranean and northeastern Atlantic ecosystems, was crustaceans, especially copepods. In general, previous investigations defined these fishes as zooplanktivorous and micronektivorous, filling the role of secondary consumers in pelagic waters [17,43,93,99,100]. Copepods were by far the most consumed organisms by myctophiforms and some stomiiforms. Nevertheless, the prevalence of small prey in the diets of some fish species in the present investigation may be partly explained by the dominance of small-sized individuals and the lack of the largest adult stages (a constraint of our sampling methodology) [101,102,103]. On the other hand, the inter-specific comparison of diet composition is more diverse when the prey items are identified to the lowest taxonomic level and expressed through different indexes (e.g., numerical abundance versus carbon content). Although some species of myctophids are considered generalists, the proportions of the ingested prey taxa varied from one species to another, and there was also a clear dietary shift throughout fish growth. Even so, our findings allow for the delineation of six trophic guilds, where some feeding characteristics and behaviors are shared at the family level, and for the comparisons of these species with the literature [45,103].

Based on the results of this study and previous investigations [36,51], the species of myctophids analyzed can be ascribed to four main trophic guilds (Table 7): zooplanktivorous-mixed crustacean feeders (*B. suborbitale* and *M. punctatum*), generalists (*L. dofleini*, *H. benoiti*, *C. maderensis*, *C. warmingii*, and *D. holti*), micronektivorous (*Lampanyctus* spp.), and copepod feeders (*B. glaciale*, *Lepidophanes gaussi*, and *N. valdiviae*). However, some species (e.g., *M. punctatum* and *H. benoiti*) showed differences in their diet composition between locations, demonstrating the feeding plasticity of the species. Additionally, the small and medium-sized classes of myctophids (mostly the transformation and juvenile stages) (*B. glaciale*, *C. maderensis*, *C. warmingii*, *D. holti*, *H. benoiti*, *L. dofleini*, *L. pusillus*, and *M. punctatum*) fed on large numbers of mesozooplankton prey, whose importance decreased as the fish grew. An exception to this trend was observed in large individuals of *D. holti* that showed an intake of more nutritious and larger prey, although in lower numbers, indicating a shift towards a more specialized diet. Species-specific feeding preferences for euphausiids were detected in large specimens of *L. crocodilus* and *S. elongatus* (89% and 91% IRI, respectively). The larger specimens of *Lampanyctus* spp. and *B. suborbitale* fed mainly on micronekton and fish (apparently myctophids or stomiiforms). Some species can decrease their migrations to upper water layers in the adult-senescent phase, as occurs in *L. crocodilus* [66], changing their diet from epipelagic crustaceans during the pelagic life stage to fish in the deeper water living stage. The myctophids *N. valdiviae* and *L. gaussi* showed similar diets that were almost exclusively based on copepods. Another investigation [46], however, considered *L. gaussi* to be a mixed crustacean feeder. In total, two major patterns of feeding can be categorized within the stomiiforms (Table 7): zooplanktivorous for small species, feeding mostly on copepods (*Cyclothone* spp., *M. muelleri*, *V. tripunctulatus*, *V. nimbaria*, and *V. attenuata*) and ostracods (*Argyropelecus* spp.) [96]; and micronektivorous for larger species (*Chauliodus* spp., *P. guernei,* and *S. elongatus*) that feed on larger and more nutritious prey such as decapods and euphausiids, which, as a consequence, are generally estimated to have a higher trophic level [37,104]. Furthermore, *C. sloani* is the only species in which copepods were absent, as it is a specialist feeder (mainly myctophids and other MFs). Moreover, not all the individuals of its population respond synchronously to diel variation in light intensity; thus, it may avoid resource competition with other fish individuals or species and contribute to its niche segregation from other co-inhabitants/mesopelagic species via depth and/or size, or through its specialized feeding on them [17,105,106]. The stomiiform species studied here that do not migrate to the near-surface layers are segregated from the myctophids during feeding hours, which prevents direct competition [99]. An interesting aspect that deserves further investigation is the presence of POM detected in the stomachs of the genera *Chauliodus* and *Cyclothone*. Finally, the contribution of fish to the diets or the exclusive piscivory of some stomiids and gonostomatids is also a relevant aspect, as they have a completely different feeding pattern to the other mesopelagic fish with a mixed diet, and might act as upper trophic level predators in the mesopelagial realm [45,107].

### 4.3. Comparison of the Diet Composition of Mesopelagic Species among Zones

Factors such as the selection of specific prey items by fish predators, the vertical distribution ranges of consumers and their prey, and the migratory behaviors of these fish may contribute to a reduction in competition for the available resources [108]. Among the myctophids studied here, *L. dofleini* showed great voracity (a high number of prey items in the stomach) and the highest diversity of prey categories in all the studied zones, revealing its non-selective and generalist feeding strategy. Additionally, *M. punctatum*, *H. benoiti*, and *C. maderensis* were quite voracious species. Specifically, *M. punctatum* attained a higher prey diversity in Atlantic waters (Lisbon), whereas *H. benoiti* and *C. maderensis* did so in the Mediterranean Sea (Balearic and Alboran zones, respectively). Nevertheless, *M. punctatum* and *H. benoiti* shared a highly similar diet composition (>66% similarity in Alboran), as did *M. punctatum* and *L. dofleini* (>40% in Lisbon). The fact that these species had a high prey diversity in their stomachs in different zones, and that they also shared a large portion of their diet compositions, suggests a certain adaptive intra-specific flexibility in their foraging behaviors (i.e., active feeding, dietary shifts depending on the regional or seasonal zooplankton availability, and generalist/facultative feeding), which might favor their survival, thus attaining them higher biomasses within myctophid communities [43,109,110,111,112]. For instance, *M. punctum* fed on calanoids, mainly from the genera *Euchaeta* and *Centropages*, in the Lisbon zone, and it preyed mainly on appendicularians in the Alboran zone. Additionally, a high prevalence of appendicularians has been reported for the specimens from the Strait of Messina in the central Mediterranean [113]. The environmental conditions in each zone may also act as a filter, preventing the competition by MFs over the same resources (the ‘niche filtering hypothesis’; [114]). Nevertheless, predator–prey interactions are inherently size-dependent, and they also act as a factor limiting the overlapping of trophic niches [42,115,116]. A clear example of this relationship is the different patterns found linked to the ontogenetic shift: *B. glaciale*, *C. maderensis*, *D. holti*, and *H. benoiti* ingested a smaller number of prey items in the smaller- and medium-sized classes than in the larger ones. Regarding the ingested number of prey, the following species deserve special mention: *C. warmingii* showed an inverse model (smaller prey items in larger fish); the largest adults of *L. dofleini* and *L. pusillus* showed a noticeable decrease in the number of prey but a feeding preference for decapods, euphausiids, larger calanoids (*Euchaeta*, *Centropages*, or *Pleuromamma*), and even fish; and the transformation individuals of *M. punctatum* were unusually voracious.

Among the stomiiforms, the non-migrant *Cyclothone* was the most abundant genus in our study [65], and the one with the highest numerical abundance in the twilight zone, although the *Cyclothone* species do not interact with the migratory species during feeding hours [7,83,117]. There was geographical segregation of some of the *Cyclothone* species studied here, i.e., *C. pygmaea* is only distributed in the Mediterranean, while *C. pallida* and *C. pseudopallida* do not occur in Mediterranean waters [60], avoiding the congeneric competition for trophic resources. This congeneric competition may also be avoided to a certain degree by vertical segregation. In the Mediterranean Sea, *C. braueri* has a shallower distribution (mainly at 400–500 m) than *C. pygmaea* (600–700 m) [65], and in the Atlantic, *C. braueri* has a shallower distribution than *C. pallida* (500–600 m), although it partially overlaps with *C. pseudopallida* (400–600 m) [82]. The diets of the *Cyclothone* species showed the lowest diversity among all the mesopelagic fish studied (small copepods and ostracods), and the number of prey consumed was usually limited to one or two (55% vacuity), except for the largest species, *Cyclothone pallida*, for which up to 26 prey items were found in the stomach. In general, the estimated amount of the diet carbon content is quite low for all the species. The other gonostomatid, *S. elongatus*, and the two sternoptychids, *A. hemigymnus* and *A. sladeni,* which feed on zooplankton, also segregate their foraging area with the near-surface migratory myctophids, as they only migrate up to 100 m (vertical spatial segregation). Some species such as *A. hemigymnus* can also feed during the daytime [47,118], thus reducing their interaction with the nocturnal mesopelagic predators while foraging. Studies based on isotopic signatures detected that the migrant fishes feeding on copepods were clearly distinguished from the partial and non-migrant copepod feeders, showing that the food web baseline differs between the epipelagic environment and deeper waters [119,120].

The carbon content contribution of the prey items to fish diets is key to better understanding pelagic ecosystems. Certain prey types can constitute a resource that is not abundantly consumed, but has a high nutritional value, likely leading to a decrease in the predator’s daily rations. Among the crustacean prey categories, the importance of each taxon in mesopelagic fish diets was contrasted when comparing their numerical abundance with their carbon mass. For instance, calanoid copepods usually dominated in the diet compositions of stomiiforms and myctophids in terms of the number of items ingested. However, larger crustaceans such as euphausiids and decapods constituted most of the estimated carbon weight in the larger-sized predators, as occurred in stomiiforms such as *C. danae* and *S. elongatus*, and myctophids such as *L. dofleini*, *D. holti*, and *Lampanyctus* spp. Another example is the migratory calanoid *Pleuromamma*, which is consumed in large numbers and constituted the major carbon bulk of myctophid copepod feeders such as *L. gaussi* and *N. valdiviae*, as well as stomiiforms such as *V. attenuata*, *V. tripunctulatus*, and *Maurolicus muelleri*. This calanoid is a vertical migrant that performs displacements all across the water column, which might increase the likelihood of prey–predator encounters. The most visible difference between the feeding patterns based on the prey carbon weight and the prey numerical biomass was found in the micronektivorous species of stomiids, and the largest adults of the *Lampanyctus* spp. These species fed on lower numbers of prey but showed a shift in the selection of large crustaceans (decapods or euphausiids) and fish with a higher carbon content. *Lampanyctus crocodilus* and *L. pusillus* from the western Mediterranean Sea preferred euphausiids [43]. In particular, the more advanced adult stages of *L. crocodilus* have been reported to decrease their migratory displacements to the epipelagic layer and remain near the benthic domain, where they feed on these larger prey [121].

The occurrence of copepods in the diet of myctophids and stomiiforms had a lower importance in terms of carbon, indicating that other prey categories were more important and contributed higher biomasses to their diets. We have mentioned the most evident difference in micronektivorous species, but other prey categories, for example, such as tunicates and other gelatinous organisms, were important in terms of their carbon weight (74% C) in the diet of *A. hemigymnus.* In this case, the tunicate-based diet of this sternoptychid is related to the peak occurrence of appendicularians in Alboran and Cadiz during the study period, although usually its diet is dominated by copepods and ostracods [26].

In most dietary studies of mesopelagic fish, the contribution of particulate organic matter (POM) could not be quantified through stomach content analysis, although its presence was evident in *Cyclothone* spp. Although we were not able to assign any carbon weight to the POM, we accounted for the number of times that the POM appeared in the diet of a species (as a “prey” individual/item itself), and thus, %N, %F, and %IRI could be obtained. Usually, POM includes detritus and marine snow, and constitutes a nutritive resource in deep ocean waters for mesopelagic fish [122,123], especially for small fishes such as *Cyclothone* spp., which play an important role in the remineralization of organic carbon at depth [117,124].

### 4.4. Influence of Zone Patterns

The environmental characteristics of each zone (e.g., the temperature, fluorescence, and zooplankton availability) influenced the fish diet composition, but other factors such as migratory behavior or individual body size might be masking the geographical influence. The highest values of fluorescence were found in Lisbon and Galicia, and to a lesser extent in Alboran. The highest temperatures were found in the two Mediterranean zones. The highest productivity of the upwelled influenced zones likely resulted in a higher zooplankton abundance, in turn enhancing the prey availability in these zones versus the more oligotrophic ones. For instance, in the productive Atlantic zones, the mesopelagics *L. dofleini* and *Vinciguerria* spp. had larger numbers of calanoid copepods, mainly represented by the genera *Centropages* and *Clausocalanus/Paracalanus*, than in the oligotrophic ones. This coincides with the results of a study during the late summer in the northwestern Atlantic off the Portuguese coast [125], where copepods were reported as forming >80% of the mesozooplankton community, with these species being among the most numerically abundant. The higher temperatures in the Mediterranean zones can be related to the presence of the cladoceran *Penilia avirostris* in the diet of several myctophids from Alboran, whereas these prey organisms were not found in Lisbon fishes.

The diet compositions (identifying prey to the lowest taxonomic level) of the myctophids from Cadiz and Lisbon were more dissimilar to each other than to those from Alboran, which overlapped with the fish diets of the Balearic and Cadiz zones. The diversity of the zooplankton from Alboran is generally higher than those in eastern parts due to the influence of the Atlantic, and might have a species composition intermediate between that of the Gulf of Cadiz and the Balearic Sea. In general terms, there were more between-species dissimilarities in the myctophid diets from the Balearic Sea, which were more similar to the diets of the species from different zones. This might indicate that fish species have a strategy to avoid competition among themselves in the Balearic Sea, as it is the most oligotrophic studied zone, and might have a lower abundance of available food resources.

The role of appendicularians in the diet also deserves comment. Appendicularians seemed important in the diet of the specimens captured in the Alboran zone (*A. hemigymnus*: 42.5% IRI; *C. maderensis*: 25% IRI; *H. benoiti*: 76% IRI; and *L. dofleini*: 23% IRI) and, to a lesser extent, in the species from the Gulf of Cadiz (*C. warmingii*: 25% IRI; and *D. holti*: 19% IRI). This fact might indicate that, during the study period, appendicularians were abundantly present in the zooplankton and were selected by some of the mesopelagic species (*C. pallida*), or captured by the generalist ones (e.g., *H. benoiti* or *C. maderensis*). Moreover, the consumption of appendicularians by these predators might suggest that they have higher trophic levels in the more productive zone of Alboran than in the oligotrophic Balearic Sea, where the baseline of the trophic web could be represented by copepods (in diets, mainly of *Pleuromamma* spp.).

Cladocerans were numerically important in *C. maderensis*, *L. dofleini*, and *H. benoiti* in Alboran, which supports the idea that these species are generalist feeders that prey on a large number of organisms that present dominant biomasses in the environment, such as cladocerans that display strong population increases under favorable conditions [79], particularly *Penilia avirostris* during the summer–autumn period in Alboran [126]. Cladocerans are epipelagic prey whose populations increase quickly under the favorable conditions of summer in the area.

There is interest in returning to classical studies based on stomach content analyses to complement isotopic and molecular data. Either of these two techniques is good for establishing numerical relationships, with the calculation of the biomass consumed by these mesopelagic species. Moreover, there are other limitations to our current knowledge of the trophic ecology of MFs, as several traits remain unevaluated (e.g., How many hours does a predator stay in the feeding zone? How many times a day does it feed? Why do the different predator species of MFs feed on different functional groups? What is driving these differences?). To allow for assumptions about the functioning of pelagic ecosystems and to define accurate trophic models, quantitative data on predatory pressure and the contribution of mesopelagic fish to carbon cycling through dietary intake are of relevance. Evaluating the predation impact of the mesopelagic assemblage on zooplankton remains an issue to be addressed, as it needs information on the individual feeding rates of species, which are nowadays mostly lacking.

The fishing grounds of the Iberian area have been intensely exploited. Mesopelagic fish are also food for commercially exploited species (e.g., tuna, hake); thus, to maintain the sustainability of fisheries’ activities, it is also essential to design strategies for the protection and sustainable assessment of mesopelagic fish communities. The present study was carried out in important fishing areas, corresponding to different geographical subareas (GSA) of the Mediterranean basin (GS5. Balearic Islands; and GS1. The Alboran Sea) and the FAO fishing subarea 27.9.a (the Gulf of Cadiz, the waters off Lisbon, and Galician waters), and thus, the information provided in this study might be potentially useful for evaluating the future protection strategies of these fishing areas.

## 5. Conclusions

Trophic studies on the mesopelagic fish community require a better understanding of species-specific feeding behaviors, well-planned experimental design, and high investment to be able to focus on the effect of the diverse factors that affect the community, since usually, there are many elements modulating mesopelagic fish feeding behavior: some species or individuals, depending on their ontogenetic development, are prey selective, or, on the contrary, are quite voracious, and consume a broad diversity of prey (e.g., *L. dofleini* and *M. punctatum*). We also found species with adaptive intra-specific flexibility in their foraging behavior (e.g., active feeding, a change of diet as a function of the regional or seasonal variability of zooplankton, and a large range of prey resources, etc.). The diet composition of generalist myctophids such as *Hygophum benoiti* and *Ceratoscopelus maderensis* reflects the different zooplanktonic communities among the zones. Moreover, geographic environmental conditions, migratory behavior, and body size are all factors that condition fish diets and allow for the identification of some major feeding patterns among these mesopelagic fish communities. The trophic niche of migrant myctophids showed a high overlap, with copepods as their primary prey category. The large stomiiforms (*Chauliodus* spp., *Stomias boa*, and *Sigmops elongatus*) preferred feeding on micronekton, while the smallest stomiiforms, *Cyclothone* spp. and *Argyropelecus* spp., preyed upon copepods and ostracods. Given the relevance of the mesopelagic fish communities to commercially exploited species and the maintenance of the sustainability of fisheries in the studied regions, the present study provides important information for a better understanding of the ecology of these species.

## Figures and Tables

**Table 1:** Summary of fish samples examined for stomach content analyses from the Mediterranean and Atlantic regions selected for the study. Migr: migratory behavior (VM: vertical migrant; PM: partial migrant; and NM: non-migrant); SL: standard length (mm); T: transformation stage; A: adult stage; and N st: number of analyzed stomachs. Sampling zones: Bal: Balearic; Alb: Alboran; Cad: Gulf of Cadiz; Lis: Lisbon; and Gal: Galicia.

Species	Migr	Region	Size Range (SL, mm)	N st	N with Prey
Stomiiformes					
Gonostomatidae					
*Cyclothone braueri*	NM	Mediterranean			
		Bal	A: 23	5	5
		Alb	A: 16.50–23.50	5	4
		Atlantic			
		Cad	A: 24–25	10	6
		Lis	A: 16–33	10	9
		Gal	A: 26–27	5	5
*Cyclothone pallida*	NM	Gal	A: 32–52	12	8
*Cyclothone pseudopallida*	NM	Atlantic			
		Cad	A: 20–35	5	4
		Lis	A: 18–26	5	5
		Gal	A: 28	1	1
*Cyclothone pygmaea*	NM	Alb	A: 18–35	5	5
*Sigmops elongatus*	PM	Atlantic			
		Cad	A: 116–140	6	6
		Lis	A: 106–125	5	4
Sternoptychidae					
*Argyropelecus hemigymnus*	PM	Mediterranean			
		Bal	T: 7–16.2	5	5
			A: 22–27	3	3
		Alb	A: 20–35	11	11
		Atlantic			
		Cad	A: 21.5–43	4	4
			T: 9–14.5	4	4
		Lis	A: 18–31	4	0
*Argyropelecus sladeni*	PM	Cad	T: 7–11	5	5
*Maurolicus muelleri*	PM	Alb	T: 13–15	4	4
			A: 16–26.5	6	6
*Valenciennellus tripunctulatus*	NM	Atlantic			
		Cad	A: 18.5–24.5	5	5
		Lis	A: 23–27.5	4	4
Phosichthyidae					
*Vinciguerria attenuata*	VM	Bal	T: 16.5–19	5	5
*Vinciguerria nimbaria*	VM	Atlantic			
		Cad	T: 15–17.5	5	5
			A: 26–33	5	5
		Lis	T: 16–23	5	4
			A: 23.5–35.5	6	6
Stomiidae					
*Chauliodus danae*	VM	Atlantic			
		Cad	T: 28.5–38	5	3
			A: 73–123	5	5
		Gal	A: 97–116	5	5
*Chauliodus sloani*	VM	Mediterranean			
		Bal	A: 61–73	3	2
		Alb	A: 200	1	1
		Atlantic			
		Cad	A: 40–113	5	5
		Lis	A: 43–123	7	7
*Photostomias guernei*	PM	Lis	A: 63–72	4	4
*Stomias boa*	VM	Cad	A: 64–123	5	2
Myctophiformes					
Myctophidae					
*Benthosema glaciale*	VM	Mediterranean			
		Bal	T: 17.5–20	5	5
			A: 22–29.5	5	5
		Alb	A: 31–32.5	5	5
*Benthosema suborbitale*	VM	Atlantic			
		Cad	A: 20–29	5	5
		Lis	A: 27	1	1
*Ceratoscopelus maderensis*	VM	Mediterranean			
		Bal	T: 16.5–18.5	5	5
			A: 21–44	5	5
*Ceratoscopelus warmingii*	VM	Atlantic			
		Cad	A: 36–42	5	5
		Lis	A: 19–29	5	5
*Diaphus holti*	VM	Mediterranean			
		Bal	A: 33–38	5	5
		Alb	T: 16–18	4	4
		Atlantic	A: 43	1	1
		Cad	A: 45–50	5	5
*Hygophum benoiti*	VM	Mediterranean			
		Bal	T: 11.5–18	10	10
			A: 19.5–20	2	2
		Alb	T: 15–19.4	8	7
*Hygophum reinhardtii*	VM	Lis	A: 20–39	5	5
*Lampanyctus alatus*	VM	Atlantic			
		Cad	A: 28–52	4	4
		Lis	A: 46–70	4	4
*Lampanyctus crocodilus*	VM	Mediterranean			
		Bal	A: 35.5, 61.5	2	2
		Alb	A: 61–84	5	5
*Lampanyctus pusillus*	VM	Mediterranean			
		Bal	T: 10, 18	2	2
			A: 10–57.5	4	4
		Alb	A: 31	1	1
		Atlantic			
		Cad	A: 25.5–33	5	5
		Lis	A: 17.5–49	6	6
*Lepidophanes gaussi*	VM	Cad	A: 18–24	5	5
*Lobianchia dofleini*	VM	Mediterranean			
		Bal	A: 30–33.5	5	5
		Alb	A: 25.5–28.5	5	5
		Atlantic			
		Cad	A: 20.5–25	5	5
		Lis	A: 25–28	4	4
*Myctophum punctatum*	VM	Mediterranean			
		Alb	A: 32–49	14	14
		Atlantic			
		Lis	A: 41.5–60	12	12
*Notolychnus valdiviae*	VM	Atlantic			
		Cad	A: 18.5–20	5	5
		Lis	A: 20, 21	2	2

**Table 2:** PERMANOVA analysis via the sum of squares Type III, with unrestricted permutations (999) of raw data. Dev: developmental stage; Migrator: migratory pattern; df: degrees of freedom; SS: mean of the sum of squares; MS: mean squares; and perm: permutations. Zone and Dev are nested in fish species.

Source	Levels	df	SS	MS	Pseudo-F-Statistic	*p* (perm)
Fish species	28	25	2.56 × 10^5^	10,250	4.6669	0.001
Zone	5	4	51,261	12,815	5.8352	0.001
Zone (fish species)		34	2.07 × 10^5^	6098.9	2.5025	0.001
Dev (fish species)	2	1	8572.6	8572.6	3.9033	0.001
Migrator	3	1	4143.3	4143.3	1.8866	0.02

**Table 3:** PERMANOVA pair-wise tests for mesopelagic fish species between zones (only significant results were listed). Bray–Curtis similarity resemblance. Sum of squares Type III. Unrestricted permutation of raw data (999). Sim %: percentage of average similarity between zones; perm: unique permutations; and cum %: cumulative percentage explained by the variable. Prey categories’ contribution to average dissimilarity percentages (% diss) considering a 90% cut-off (similarity percentage analysis, SIMPER) is shown on the right side of the table.

Groups	Sim %	*t*	P	perm	Cum %	Prey Categories (% diss)
***A. hemigymnus*** Alboran, Cadiz	14.71	1.88	0.03	35	57	Appendicularia (18); Calanoida (13); *Clausocalanus* + *Paracalanus* (10); *Conchoecia* (9)
***A. hemigymnus*** Balearic, Alboran	14.79	1.85	0.007	312	58	Appendicularia (23); Calanoida (14); *Conchoecia* (11); *Clausocalanus* + *Paracalanus* (10)
***C. braueri*** Lisbon, Galicia	0	1.93	0.046	12	57	*Oncaea* (22); Calanoida (21); POM (14)
***C. maderensis*** Balearic, Alboran	12.55	1.77	0.016	123	53	Appendicularia (21); Copepoda (12); *Pleuromamma* (11); *Conchoecia* (9)
***C. warmingii*** Cadiz, Lisbon	8.4	2.05	0.01	126	56	*Centropages* (17); *Conchoecia* (12); Appendicularia (10); Calanoida (9); *Pleuromamma* (6)
***D. holti*** Balear, Cadiz	19.5	1.83	0.012	126	52	*Conchoecia* (16); *Pleuromamma* (11), *Oncaea* (9); Euphausiacea (8); Chromista (8)
***D. holti*** Alboran, Cadiz	35.6	1.88	0.008	126	57	*Conchoecia* (13); *Pleuromamma* (12); *Oncaea* (12); Chromista (10); Appendicularia (10)
***H. benoiti*** Balearic, Alboran	23.59	2.05	0.001	987	54	Appendicularia (36); *Oncaea* (7); Corycaeidae (6); *Evadne* + *Podon* (5)
***L. dofleini*** Alboran, Cadiz	39.38	2.02	0.005	126	53	Appendicularia (12); *Centropages* (12); Calanoida (8); Corycaeidae (7); *Conchoecia* (6); *Penilia* (6)
***L. dofleini*** Balearic, Cadiz	33.78	2.23	0.008	126	50	*Oncaea* (16); Corycaeidae (14); Euphausiacea (11); *Conchoecia* (9)
***L. dofleini*** Alboran, Lisbon	37.8	1.42	0.024	126	35	Appendicularia (14); *Centropages* (8); Calanoida (7); *Penilia* (6)
***L. dofleini*** Balear, Lisbon	26.38	1.84	0.006	126	38	Calanoida (9); Euphausicea (8); *Centropages* (8); *Calanus* (7); *Candacia* (7); Larval Euphausiacea (5); *Candacia* (5); *Calanus* (5)
***L. dofleini*** Cadiz, Lisbon	29.33	2.28	0.01	126	57	Corycaeidae (13); *Conchoecia* (12); *Oncaea* (12); Calanoida (7); *Centropages* (7); Appendicularia (6)
***L. dofleini*** Balearic, Lisbon	39.67	1.79	0.006	126	52	Calanoida (9); Euphausiacea (8); *Centropages* (8); *Calanus* (7); *Candacia* (7); *Pleuromamma* (7); *Conchoecia* (6)
***M. punctatum*** Alboran, Lisbon	17.53	5.85	0.001	999	51	Appendicularia (21); *Centropages* (11); *Euchaeta* (10); Calanoida (9)
***V. tripunctulatus*** Cadiz, Lisbon	5.04	3.4	0.009	163	61	*Pleuromamma* (29), Calanoida (12), *Conchoecia* (11), *Centropages* (10)

**Table 4:** PERMANOVA pair-wise tests between developmental stages (groups: transformation and adult including the juvenile stage) and within species. Only significant pair-wise tests are listed. Bray–Curtis similarity resemblance. Sum of squares Type III. Unrestricted permutation of raw data (999). *t*: *t*-statistic; df: degrees of freedom; perm: permutations; sim %: percentage of average similarity between and within groups; and prey categories’ contribution to average dissimilarity percentages (% diss) considering a 90% cut-off (similarity percentage analysis, SIMPER) is shown on the right side of the table.

Fish Species	Groups	*t*	df	*p* (perm)	Sim %	Prey Cat (% Diss)	
*A. hemigymnus*	T, A	1.61	11	0.045	T: 63.9; A: 30.9 T, A: 34.1	Transformation	
						Ostracoda	67.5
						Non-Calanoida	30.1
						Adult	
						Ostracoda	53.5
						Appendicularia	19.1
*H. benoiti* A	T, A	1.75	15	0.01	T: 40.9; A: 38.2 T, A: 30.0	Transformation	
						*Oncaea*	53.8
						Calanoida	22.2
						Appendicularia	15.1
						Adult	
						*Pleuromamma*	55.1
						*Oncaea*	45.1

**Table 5:** Main contribution of prey categories to the dissimilarity between the diets of migrant and non-migrant mesopelagic fish (similarity percentage analysis, SIMPER). Values in the columns “M” and “NM” are average abundances of the migrant and non-migrant groups, respectively; Av Diss: average dissimilarity; SD: Standard deviation; and Contr: percentage of prey contribution to diet composition.

Species	M	NM	Av Diss	SD	Contr (%)
*Pleuromamma*	0.78	0.33	12.55	0.85	14.75
Non-Calanoida	1.14	0.34	12.39	0.91	14.56
Other Calanoida	1.23	0.32	11.16	0.90	13.11
Eucarida (euphausiids and decapods)	0.36	0.27	8.70	0.61	10.22
Ostracoda	0.44	0.24	7.61	0.66	8.94
Appendicularia	1.16	0.00	7.18	0.50	8.44
*Centropages*	0.63	0.05	5.24	0.52	6.16
*Euchaeta*	0.44	0.14	4.70	0.50	5.52
*Candacia* spp. and *Acartia* spp.	0.36	0.05	4.24	0.46	4.98
Gelatinous	0.14	0.16	3.48	0.35	4.08

**Table 6:** Canonical correspondence analysis for the studied mesopelagic species and the environmental variables SST (sea surface temperature), T450 (temperature at a 450 m depth), and FI (integrated fluorescence). Var (%) is the proportion of variance explained for each variable. P is the significance for each variable. Perms is the number of permutations and inter-set correlations of environmental variables with the first two axes.

Species		Conditional Effects			Inter-Set Correlations	
Variable	Var (%)	*p*	Perms	F	Axis 1	Axis 2
T450	12.6	0.001	999	3.82	−0.6839	−0.1812
SST	11.3	0.003	999	2.96	−0.5571	0.3512
Fl	11.2	0.15	999	1.41	0.5537	0.3547

**Table 7:** Trophic guilds defined for the studied species are presented in combination with their migratory behavior and feeding patterns obtained from the estimation of prey carbon weight and prey abundance. The last two columns correspond to the guilds defined in previous studies [36,51]. M: migrant; PM: partial migrant; and NM: non-migrant. * Predominance of the prey category indicated. ** The species analyzed in the study was the congener *L. guentheri*, not *L. gaussi*.

Species	Trophic-Migratory Guilds	Feeding Patterns Based on		Hopkins, Sutton, Lancraft (1996) [51]	Czujaz et al. (2020) [36]
		Prey Carbon Weight	Count of Prey Items		
*Argyropelecus hemygimnus*	Zooplanktivore PM	Gelatinous, crustaceans	Mixed crustacean feeder	Not analyzed	Not analyzed
*Argyropelecus sladeni*	Zooplanktivore PM	Mixed crustacean feeder	Mixed crustacean feeder	Not analyzed	Not analyzed
*Maurolicus muelleri*	Zooplanktivore M	Mixed crustacean feeder	Copepod feeder 71% *	Small crustaceans *	Not analyzed
*Valenciennellus tripunctulatus*	Zooplanktivore PM	Mixed crustacean feeder	Copepod feeder 77% *	Small crustaceans *	Not analyzed
*Chauliodus danae*	Micronektivore M	Decapod feeder	Mixed crustacean, fish, micronekton, POM	Piscivorous	Not analyzed
*Chauliodus sloani*	Micronektivore M	Piscivorous	Micronektivore	Piscivorous	Not analyzed
*Photostomias guernei*	Micronektivore PM	Other minor categories	Euphausiida, Decapoda	Decapod feeder	Not analyzed
*Stomias boa*	[Two guts with prey]	Gelatinous	Gelatinous	Not analyzed	Not analyzed
*Cyclothone braueri*	Zooplanktivore NM	Copepods (*Pleuromamma, Euchaeta*) *	Copepod feeder 60% *	Copepod 72% *	Not analyzed
*Cyclothone pallida*	Gelatinous feeder NM	Gelatinous and copepods	Gelatinous (Tunicata)	Copepod 72% *	Not analyzed
*Cyclothone pseudopallida*	Zooplanktivore NM	Mixed crustacean feeder	Copepod feeder	Copepod 72% *	Not analyzed
*Cyclothone pygmaea*	Zooplanktivore NM	Small copepod feeder	Non-defined	Not analyzed	Not analyzed
*Sigmops elongatus*	Micronektivore PM	Euphausiid feeder	Euphausiid 61% *	Euphausiids 40% *	Mixed crustacean NM
*Vinciguerria attenuata*	Zooplanktivore M	Mixed crustacean feeder	Mixed crustacean feeder	Small crustaceans	Not analyzed
*Vinciguerria nimbaria*	Zooplanktivore M	Mixed crustacean feeder	Mixed crustacean feeder	Crustaceans, fish	Copepod feeders PM
*Benthosema glaciale*	Zooplanktivore M	Mixed crustacean feeder	Mixed crustacean feeder	Not analyzed	Not analyzed
*Benthosema suborbitale*	Zooplanktivore M	Mixed crustacean feeder	Micronektivorous	Small crustaceans	Not analyzed
*Ceratoscopelus maderensis*	Generalist M	Crustaceans, gelatinous, fish	Crustaceans and gelatinous 55% *	Not analyzed	Copepod feeder M
*Ceratoscopelus warmingii* (Atl)	Generalist M	Mixed crustacean feeder	Crustaceans and gelatinous 20% *	Gelatinous 37% *	Copepod feeder M
*Diaphus holti*	Generalist M	Euphausiid feeder	Mixed crustacean and gelatinous feeder	Not analyzed	Not analyzed
*Hygophum benoiti* (Med)	Generalist M	Mixed crustacean and gelatinous feeder	Mixed crustacean and gelatinous feeder	Small crustaceans *	Not analyzed
*Hygophum reindhardtii*	Zooplanktivore M	Mixed crustacean feeder	Mixed crustacean feeder	Mixed crustacean *	Not analyzed
*Lampanyctus alatus*	Micronektivore M	Euphausiid	Mixed crustacean feeder	Mixed crustacean	Not analyzed
*Lampanyctus crocodilus*	Micronektivore M	Piscivorous	Micronektivore	Not analyzed	Not analyzed
*Lampanyctus pusillus*	Micronektivore M	Euphausiid feeder 60%	Copepod feeder >80%	Not analyzed	Not analyzed
*Lobianchia dofleini*	Generalist M	Mixed crustacean feeder (copepods, euphausiids)	Mixed crustacean and gelatinous feeder	Not analyzed	Not analyzed
*Lepidophanes gaussi* (Atl)	Copepod feeder M	Copepod feeder (*Pleuromamma, Candacia*)	Mixed crustacean feeder	Mixed crustacean feeder	Copepod feeder M **
*Myctophum punctatum*	Zooplanktivore M	Mixed crustacean, gelatinous feeder	Gelatinous and copepod feeder	Not analyzed	Not analyzed
*Notolychnus valdiviae* (Atl)	Copepod feeder M	Copepod feeder	Copepod feeder	Small crustaceans *	Not analyzed

## Data Availability

The raw data supporting the conclusions of this article will be made available by the authors, without undue reservation.

## Supplementary Materials

The following supporting information can be downloaded at: https://www.mdpi.com/article/10.3390/ani13050886/s1. Table S1: Length–weight relationships for specific prey taxa and their carbon mass estimations as a percentage of body dry weight. Source indicates the bibliographic reference used for the calculation of DM and CM. Locations 1 and 2 refer to the geographic areas of study, of the literature cited, for DM and CM relationships, respectively. DM: dry mass; CM: carbon mass; WM: wet mass; L: body length; SL: standard length; TL: total length and trunk length in the case of appendicularians; V: volume; W: body width. Own data: in this case, the measurements obtained from mesopelagic fish from previous survey cruises (Migrants and Active Flux in the Atlantic Ocean project, MAFIA; CTM2012-39587-C04-03)) [98,127,128,129,130,131,132,133,134,135,136,137,138,139,140,141,142,143,144,145,146,147,148,149,150,151,152], Table S2: Diet composition of myctophids and stomiiforms in terms of prey number, abundance (%N), frequency of occurrence (%F), and index of relative importance (%IRI). Cells with zeros indicate the absence of data, Table S3: Mean Shannon diversity index (H’) and mean prey number per fish species.
