# Supplementary material for: Multiple Approaches to the Trophic Role of Mesopelagic Fish around the Iberian Peninsula
Source: Animals (Basel). 2023 Feb 28;13(5):886. doi: 10.3390/ani13050886 (PMC10000212; doi:10.3390/ani13050886)
Supplement: Supplementary file 1 [file animals-13-00886-s001.zip › animals-2020174-supplementary Final/Supplementary Materials.pdf]

animals

MDPI

Table S1. Length–weight relationships for specific prey taxa and their carbon mass estimations as a percentage of body dry weight. Source indicates the bibliographic reference used for the calculation of DM and CM. Locations 1 and 2 refer to the geographic areas of study, of the literature cited, for DM and CM relationships, respectively. DM: dry mass; CM: carbon mass; WM: wet mass; L: body length; SL: standard length; TL: total length and trunk length in the case of appendicularians; V: volume; W: body width. Own data: in this case, the measurements obtained from mesopelagic fish from previous survey cruises (Migrants and Active Flux In the Atlantic Ocean project, MAFIA; CTM2012-39587-C04-03)).

Taxa	Length DM Equation	Source DM	Location 1	CM (%DM)	Length CM Equation	Source CM	Location 2
Amphipoda	$DM_{\mu g} = 10^{0.69} \times L_{mm}^{2.72}$	[127]	Subarctic Pacific	30.0		[128]	NW Mediterranean
Cladocera	$Log_{10}DM_{\mu g} = 3.90 \times log_{10}L_{\mu m} - 10.12$	[129]	Sea of Japan	33.1		[128]	NW Mediterranean
Podon	$Log_{10}DM_{\mu g} = 3.90 \times log_{10}L\mu m - 10.12$	[129]	Sea of Japan	33.1		[128]	NW Mediterranean
Penilia	$Log_{10}DM_{\mu g} = 4.99 \times log_{10}L_{\mu m}-13.77$	[129]	Sea of Japan		$LogC_{\mu g} = 4.51 \times LogL_{\mu m} - 12.74$	[129]	Sea of Japan
Calanoida	$DM_{\mu g}=7.5 \times 10^{-8} L_{\mu m}^{2.74}$	[130]	Caribean Atlantic	42.4		[131]	Southern Benguela
Acartiidae	$Log_{10}DM_{\mu g} = Log_{10}L_{\mu m}\ 2.97 - 7.72$	[132]	North Sea	37.3		[98]	NW Mediterranean
Candacia	$DM_{\mu g} = 1.275 \times 10^{-9} \times L_{\mu m}^{3.55}$	[133]	Caribean Atlantic	36.6		[128]	NW Mediterranean
Centropages	$In\ DM_{\mu g} = 3.68 \times lnL_{\mu m} - 22.86$	[130]	Caribean Atlantic	28.2		[98]	NW Mediterranean
Euchaeta	$DM_{\mu g} = 7.5 \times 10^{-8} \times L_{\mu m}^{2.74}$	[130]	Caribean Atlantic	36.0		[98]	NW Mediterranean
Pleuromamma	$DM_{\mu g} = 7.5 \times 10^{-8} \times L_{\mu m}^{2.74}$	[130]	Caribean Atlantic	30.1		[98]	NW Mediterranean
Temora	$Log_{10}DM_{\mu g} = Log_{10}L_{\mu m}\ 3.064 - 7.6958$	[134]	Andaman Sea	37.3		[98]	NW Mediterranean
Nauplius	$DM_{mg} = 0.0173 \times L_{mm}^{2.27}$	[135]	North Sea	40.0		[135]	North Sea
Cyclopoida	$DM_{\mu g} = 8.807 \times 10^{-6} L_{\mu g}^{1.96}$	[130]	Caribean Atlantic	44.8		[136]	Subarctic Pacific
Corycaeidae	$Log_{10}DM_{\mu g} = 1.997 \times Log_{10}L_{\mu m} - 5.3245$	[137]	Subarctic Pacific	33.3		[98]	NW Mediterranean
Oncaeidae	$In\ DM_{\mu g} = 2.90 \times In\ (L_{\mu m}) - 16.81$	[133]	Andaman Sea	29.5		[98]	NW Mediterranean
Harpacticoida	$DM_{\mu g} = 1.389 \times 10^{-8} L_{\mu m}^{2.857}$	[138]	Brazilian estuary	46.4		[138]	Brazilian estuary
Microsetella type	$In\ DM_{\mu g} = 1.15 \times In\ (L_{\mu m}) - 7.10$	[133]	Andaman Sea	50.2		[133]	Andaman Sea
Ovisac copepod	1.1 µg			40.0		[139]	

Copepoda	$DM_{\mu g} = 7.5 \times 10^{-8}\ L_{\mu m}^{2.74}$	[130]	Caribean Atlantic	42.4		[131]	Southern Benguela
Egg					$CM\mu g = 160 \frac{4}{3} \pi \left(\frac{L_{mm}}{2}\right)^3$	[140]	Subtropical estuary (Florida), temperate shelf waters (California), Irish Sea). and the Gulf Stream
Euphausiacea (larva)	$Log_{10}WM_{\mu g} = log_{10}TL_{mm}^{3.13} + 0.914$; DM = 21% WM	[137]		35.6		[98]	NW Mediterranean
Euphausiacea (adult)	$DM_{mg} = 0.0012\ TL_{mm}^{3.16}$	[131]		35.6		[128]	NW Mediterranean
Decapoda	$Log_{10}DM_{mg} = (2.755\ Log_{10}L_{mm} - 1.6693) + Log_{10}(0.25)$			40.0		[128]	NW Mediterranean
Malacostraca	$Log_{10}DM_{mg} = (2.755\ Log_{10}L_{mm} - 1.6693) + Log_{10}(0.25)$			40.0		[128]	NW Mediterranean
Ostracoda	$Log_{10}DM_{mg} = 2.42\ Log_{10}L_{mm} - 1.34$	[141]	Weddell Sea	27.3		[98]	NW Mediterranean
Polychaeta	$DMmg=0.005\ L_{mm}^{2.25}$	[142]	W Norway	52.7		[129]	Sea of Japan
Chaetognatha	$Log_{10}DM_{\mu g} = 2.91\ Log_{10}TL_{mm} - 0.79$	[143]	WN Pacific Ocean	29.0		[129]	Sea of Japan
Mollusca							
Gastropoda	$DM_{mg} = 0.1365\ L_{mm}^{1.501}$	[144]	Southern Ocean	25.6		[128]	NW Mediterranean
Bivalvia	$DM_{mg} = 0.1365\ L_{mm}^{1.501}$			17.7		[129]	Sea of Japan
Cephalopoda beak	250 µg	Own calculation		40.0		[145]	
Cnidaria	$Log_{10}DM_{\mu g} = 2.94\ Log_{10}TL_{mm} + 0.82$	[143]	WN Pacific Ocean	23.4		[128]	NW Mediterranean
Tintinnida; Ciliophora					$CM_{\mu g} = 1.4\ x10^{-7x}\ V_{\mu m^3}$	[146]	Estuarine Coastal waters
Radiolaria					$CM_{\mu g} = 2.6E - 10 \times V_{\mu m^3}$	[147]	
Foraminifera					$CM_{\mu g} = 1.8E - 8 \frac{4}{3} \pi \left(\frac{L_{\mu m}}{2}\right)^3$	[147]	
Phytoplankton					$CM_{pg} = 0.288 \times V(_{\mu m^3})^{0.811}$	[148]	

Fecal pellet					$CM_{\mu g} = (0.01\ (4/3)\ \pi\ (W_{\mu m})^2\ L_{\mu m}))\ x\ 10^{-6}$	[149]	Laboratory
Appendicularia	$DW_{\mu g} = 11.3\ x\ TL_{mm}^{1.77}$	[150]	Newfoundland (Canada)	40.0	$CM_{\mu g} = 1.445\ x\ 10^{-7}\ x\ TL_{\mu m}^{2.59}$	[98]	NW Mediterranean
Thaliacea; Salpida					$CM_{\mu g} = 1.62\ x\ TL_{mm}^{1.93}$	[151]	South Pacific
Actinopterygii (larva)	250 μg			40.0		Conservative estimation from own data	
Actinopterygii	$DM_{mg} = 0.00133\ x\ SL_{mm}^{3.1502}$	Own data	Central Atlantic	40.0		Own data	
Pollen	0.0013 μg			52.0		[152]	

Table S2. Diet composition of myctophids and stomiiforms in terms of prey number, abundance (%N), frequency of occurrence (%F), and index of relative importance (%IRI). Cells with zeros indicate the absence of data.

Table S3. Mean Shannon diversity index (H′) and mean prey number per fish species.

**Fish Species**	**H′**						**Number of Prey Items**		
	**Alb**	**Bal**	**Cad**	**Gal**	**Lis**	**H′ Total**	**Mean PN**		**SD**
**Stomiiformes**									
Cyclothone braueri		0.7	0.7	0.7	0.6	0.7	A	2	0.88
C. pallida				0.3		0.3	A	26	0
C. pseudopallida							A	1	0.79
C. pygmaea							A	1	0
Sigmops elongatus			1.0			1.0	A	2	1.05
Argyropelecus hemygimnus	1.4	1.1	1.0		0.8	1.1	T	6	4.27
							A	13	9.92
A. sladeni			1.0			1.0	T	3	1.82
Maurolicus muelleri	1.4					1.4	T	13	3.30
							A	12	6.09
Valenciannelus tripunctulatus			1.2		0.7	1.0	A	6	3.16
Vinciguerria attenuata		1.4				1.4	T	11	12.36
V. nimbaria			1.5		1.4	1.4	T	13	11.25
							A	19	39.00
Chauliodus danae			0.7			0.7	T	2	1.00
							A	1	0.41
Chauliodus sloani		1.5				1.5	A	2	2.96
Photostomias guernei							A	2	0.82
Stomias boa							A	2	0

**Myctophiformes**								
Ceratoscopelus maderensis	1.5	1.1			1.2	T	2	1.53
						A	19	26.86
Benthosema glaciale	1.1				1.1	T	2	0.96
						A	4	3.67
Benthosema suborbitale			1.2	1.8	1.3	A	7	5.75
Ceratoscopelus warmingii			1.2	1.1	1.2	A	7	3.07
Diaphus holti	1.0	1.2	1.6		1.2	T	5	2.63
						A	9	6.10
Hygophum benoiti	0.7	1.4			1.1	T	47	55.67
						A	19	2.12
H. reinhardtii				1.0	1.0	A	5	1.00
Lampanyctus alatus			0.6		0.6	A	2	1.30
Lobianchia dofleini	2.0	1.5	1.7	1.8	1.7	A	29	28.18
Lepidophanes guernei			1.1		1.1	A	4	3.36
Lampanyctus pusillus	1.1	0.7	1.0	1.2	1.1	A	4	2.77
Myctophum punctatum	1.1			1.7	1.4	A	123	116.49
Notolychnus valdiviae				0.7	0.7	A	2	0.79
